# Small RNA profiling for identification of microRNAs involved in regulation of seed development and lipid biosynthesis in yellowhorn

**DOI:** 10.1186/s12870-021-03239-4

**Published:** 2021-10-12

**Authors:** Li Wang, Chengjiang Ruan, Aomin Bao, He Li

**Affiliations:** 1 Key Laboratory of Biotechnology and Bioresources Utilization, Ministry of Education, Institute of Plant Resources, Dalian Minzu University, Dalian, 116600 China; 2 Key Laboratory of Marine Genetics and Breeding (OUC), Ministry of Education, College of Marine Life Science, Ocean University of China, Qingdao, 266100 China; 3 Institute of Economic Forest, Tongliao Academy of Forestry Science and Technology, Tongliao, 028000 China

**Keywords:** Yellowhorn, MicroRNA, Lipid biosynthesis, Seed development, Target gene

## Abstract

**Background:**

Yellowhorn (*Xanthoceras sorbifolium*), an endemic woody oil-bearing tree, has become economically important and is widely cultivated in northern China for bioactive oil production. However, the regulatory mechanisms of seed development and lipid biosynthesis affecting oil production in yellowhorn are still elusive. MicroRNAs (miRNAs) play crucial roles in diverse aspects of biological and metabolic processes in seeds, especially in seed development and lipid metabolism. It is still unknown how the miRNAs regulate the seed development and lipid biosynthesis in yellowhorn.

**Results:**

Here, based on investigations of differences in the seed growth tendency and embryo oil content between high-oil-content and low-oil-content lines, we constructed small RNA libraries from yellowhorn embryos at four seed development stages of the two lines and then profiled small RNA expression using high-throughput sequencing. A total of 249 known miRNAs from 46 families and 88 novel miRNAs were identified. Furthermore, by pairwise comparisons among the four seed development stages in each line, we found that 64 miRNAs (53 known and 11 novel miRNAs) were differentially expressed in the two lines. Across the two lines, 15, 11, 10, and 7 differentially expressed miRNAs were detected at 40, 54, 68, and 81 days after anthesis, respectively. Bioinformatic analysis was used to predict a total of 2654 target genes for 141 differentially expressed miRNAs (120 known and 21 novel miRNAs). Most of these genes were involved in the fatty acid biosynthetic process, regulation of transcription, nucleus, and response to auxin. Using quantitative real-time PCR and an integrated analysis of miRNA and mRNA expression, miRNA-target regulatory modules that may be involved in yellowhorn seed size, weight, and lipid biosynthesis were identified, such as miR172b-*ARF2* (*auxin response factor 2*), miR7760-p3_1-*AGL61* (*AGAMOUS-LIKE 61*), miR319p_1-*FAD2–2* (*omega-6 fatty acid desaturase 2–2*), miR5647-p3_1-*DGAT1* (*diacylglycerol acyltransferase 1*), and miR7760-p5_1-*MED15A* (*Mediator subunit 15a*).

**Conclusions:**

This study provides new insights into the important regulatory roles of miRNAs in the seed development and lipid biosynthesis in yellowhorn. Our results will be valuable for dissecting the post-transcriptional and transcriptional regulation of seed development and lipid biosynthesis, as well as improving yellowhorn in northern China.

**Supplementary Information:**

The online version contains supplementary material available at 10.1186/s12870-021-03239-4.

## Background

Yellowhorn (*Xanthoceras sorbifolium*), a member of the Sapindaceae family, is endemic to northern China. This woody oil-bearing species is widely distributed throughout its native range and grows well in barren lands with a dry climate [1, 2]. Its seed kernels (embryos) can contain as much as 67% oil content, of which unsaturated fatty acids (FAs) make up to 85–93%. These FAs include linoleic acid (37.1–46.2%), oleic acid (28.6–37.1%), and nervonic acid (1.3–3.1%) [3, 4], all of which are considered beneficial to human health. Yellowhorn has attracted considerable interest in recent years due to the potential food and medicinal value of its seed oil. However, the seed oil content varies significantly among yellowhorn varieties [5]. To achieve maximum oil yield, a better understanding of the key transcriptional regulatory sites involved in seed development and lipid biosynthesis metabolic pathways is necessary. Investigating the mechanisms of regulation of seed development and lipid biosynthesis in yellowhorn is therefore of practical significance.

Yellowhorn research over the past decade has focused on oil extraction, FA composition, and the use of seed oil as a biodiesel [5–7]. Yellowhorn lipid biosynthesis metabolic pathways have been the subject of several studies, which identified some key genes associated with oil accumulation [8, 9]. For example, two novel *diacylglycerol acyltransferase* (*XsDGAT1* and *XsDGAT2*) yellowhorn genes were found to control its seed oil content [10]. In addition, de novo transcriptome analysis of multiple yellowhorn tissue types identified lipid biosynthesis and metabolism-related pathways and genes [11]. High- and low-oil yellowhorn embryo tissues at four different developmental stages were analyzed using comparative RNA-Seq analysis and it was found that many genes played critical roles in promoting oil accumulation, including several transcription factors as well as genes involved in FA biosynthesis, glycolysis/gluconeogenesis, and pyruvate metabolism [12]. The yellowhorn genome sequence was recently sequenced and assembled [13, 14]; besides, based on a large number of single-copy orthologous genes shared by 11 plant genomes, phylogenetic analysis suggested that yellowhorn and longan (*Dimocarpus longan*) in the Sapindaceae family showed the closest relationship, and diverged from their most recent common ancestor ∼33.07 million years ago [13]. In summary, research focused on the identification of lipid biosynthesis-related genes in yellowhorn has been limited. The regulatory mechanisms involved in seed development and lipid biosynthesis at the post-transcriptional levels (e.g., microRNAs) remain unknown.

MicroRNAs (miRNAs) are small, endogenous, non-coding RNAs produced from stem-loop precursors by Dicer-catalyzed excisions. MiRNAs direct RNA cleavage or block translation of target transcripts to regulate gene expression post-transcriptionally [15]. A large body of research has shown that miRNAs are critical to diverse aspects of biological and metabolic processes in seeds, including embryogenesis, pattern establishment, and lipid metabolism [16–18]. Zhang et al. [19] found that silencing miR398 in rice can increase panicle length, grain number, and grain size. Thirteen miRNAs were found to regulate oleic acid accumulation in safflower through the deep sequencing of small RNA libraries [20], and 30 miRNAs were found to regulate lipid metabolism in *Camelina sativa* [21]. Research has determined that many miRNAs control genes that function in FA biosynthesis and accumulation [22, 23]. The miR156 family, for example, directly participates in the regulation of FA biosynthesis in *Brassica napus* through its targeting of *3-oxoacyl-ACP reductase* (*KAR*) [24], and miR159b, with its targets *omega-6 fatty acid desaturase 2* (*FAD2*), *fatty acid elongase 1* (*FAE1*), and *fatty acyl-ACP thioesterase B* (*FATB*), can affect the levels of oleic acid, palmitic acid, and eicosenoic acid in *Arabidopsis thaliana* seeds [25]. In addition, the regulatory module miR167A-*ARF8* (*auxin response factor 8*) has been found to affect the α-linolenic acid content and seed size in *C. sativa* [26]. Taken together, this body of evidence indicates that miRNAs have diverse biological functions related to seed development and lipid biosynthesis in many plant species. However, the biological information of miRNAs of yellowhorn is currently limited, and this research remains in the initial stage. Previous reports have mainly focused on the effects of miRNA on floral organ development and sex differentiation [27–29]. Even so, the miRNA-mediated regulatory networks that determine seed development and oil accumulation are poorly understood in the woody oil crop yellowhorn.

In the present study, yellowhorn embryos at four seed developmental stages (40, 54, 68, and 81 days after anthesis (daa) from high-oil-content (HO) and low-oil-content (LO) lines were used to construct small RNA (sRNA) libraries. The miRNA expression was then profiled using high-throughput sequencing. In total, 249 known miRNAs and 88 novel miRNAs were identified in yellowhorn. Computational analysis was used to detect differentially expressed miRNAs among different developmental stages and among embryos from high- and low-oil lines. In addition, an association analysis between miRNA and mRNA expression was conducted to elucidate the regulatory relationship between miRNAs and their corresponding mRNA targets. This analysis was studied to understand seed development and lipid biosynthesis in yellowhorn.

## Results

### Changes in the oil content, seed size, and weight at different seed developmental stages

**Fig. 1:**
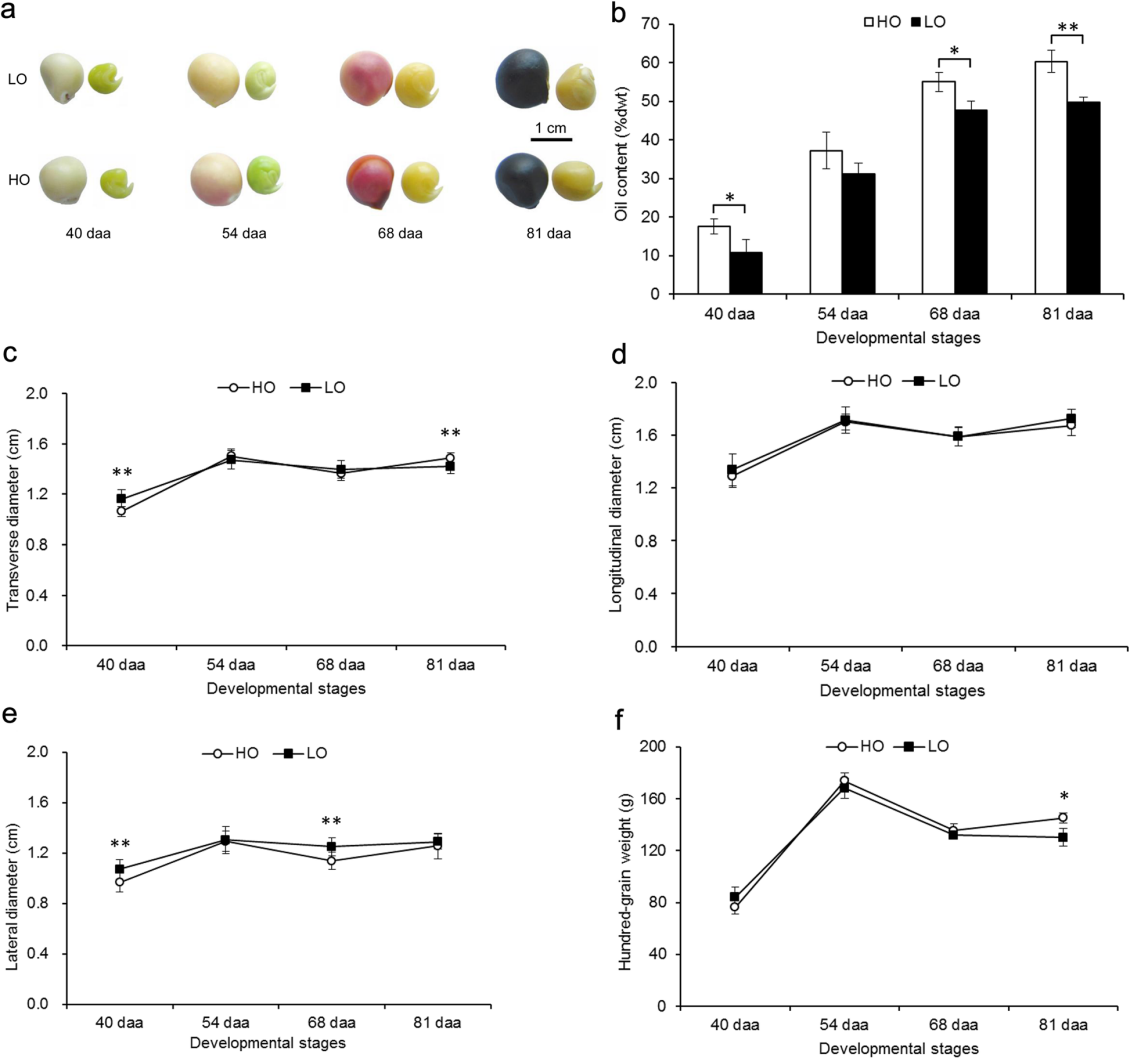
The embryo oil content, size, and weight of seeds from the two yellowhorn lines at different developmental stages. **a** Seeds and embryos of yellowhorn at four development stages. (Picture cited from Wang et al. [12]). **b** Embryo oil contents in seeds from the two yellowhorn lines at four development stages. (Data from Wang et al. [12]). **c** Transverse diameter of HO and LO seeds at four developmental stages. **d** Longitudinal diameter of HO and LO seeds at four developmental stages. **e** Lateral diameter of HO and LO seeds at four developmental stages. **f** Hundred-grain weight of HO and LO seeds at four developmental stages. Error bars indicate standard deviations for three biological replicates. ** and * indicate significant differences between the lines at the same developmental stage based on a Student’s T-test at *P* < 0.01 and *P* < 0.05, respectively

The embryo oil content, seed size, and weight were examined at four seed developmental stages (40, 54, 68, and 81 daa) in the HO and LO lines. Images of yellowhorn seeds and embryos were produced at four seed developmental stages for comparison (Fig. 1a). Both lines showed a rapid accumulation of the embryo oil content from 40 to 68 daa followed by a very slow increase from 68 to 81 daa; the highest oil content was produced at 81 daa (Fig. 1b). In both yellowhorn lines, the oil content accumulated at the highest rate between 40 and 54 daa, indicating that substantial lipid biosynthesis mainly occurred during the early to middle stages of seed development. There was significantly higher oil content in the HO embryos than in the LO embryos at 40, 68, and 81 daa, but not at 54 daa. At 81 daa, the oil content of HO embryos was 10.69% higher than that of LO (Fig. 1b).

Additionally, the transverse diameters, longitudinal diameters, and lateral diameters of the seeds increased in both lines from 40 to 54 daa and then decreased at 68 daa followed by a slow increase from 68 to 81 daa, indicating that faster growth of developing seeds occurred mainly at the early stages (40–54 daa) (Fig. 1c–e). At 54 daa, the seed sizes of the two lines were at the highest level. Moreover, the transverse diameters and lateral diameters of the seeds from the HO line were significantly smaller than those from the LO line at 40 daa, and the lateral diameters of the seeds from the HO line were significantly smaller than those from the LO line at 68 daa (Fig. 1c, e). With seed development, the change in the weight of the two lines was similar to the change of the seed size, and the HO line had a higher hundred-grain weight than the LO line (Fig. 1f).

### Overview of sRNA sequencing in yellowhorn

Sixteen sRNA libraries from eight samples (two biological replicates for each) were constructed to identify miRNAs linked to the regulation of oil accumulation and seed development. A total of 238,325,077 raw reads were obtained from these libraries by high-throughput sequencing (approximately 14.9 million raw reads per library). A total of 194,958,060 clean reads of 18–25 nucleotides (nt) and an average of 12,184,879 reads (81.80%) per library were obtained after removing the adaptor dimers, junk reads, low-complexity sequences, and sequences shorter than 18 nucleotides and longer than 25 nucleotides (Table 1).

**Table 1:** Summary of small RNA sequencing statistics

Sample		Raw reads	Adaptor sequence and length with < 18 nt and > 25 nt	Junk reads	Clean reads
HO40	HO40_1	17,087,566	1,824,397	160,613	15,102,556
	HO40_2	15,381,430	1,191,349	131,834	14,058,247
HO54	HO54_1	15,504,184	1,464,666	147,468	13,892,050
	HO54_2	18,792,450	2,046,587	134,962	16,610,901
HO68	HO68_1	12,770,282	1,373,111	82,560	11,314,611
	HO68_2	13,660,269	4,816,480	30,860	8,812,929
HO81	HO81_1	13,489,436	2,151,260	62,326	11,275,850
	HO81_2	10,989,687	2,342,466	74,391	8,572,830
LO40	LO40_1	14,051,494	2,970,987	122,646	10,957,861
	LO40_2	13,797,449	4,423,308	93,188	9,280,953
LO54	LO54_1	14,790,646	3,030,592	54,704	11,705,350
	LO54_2	14,346,000	2,721,911	108,500	11,515,589
LO68	LO68_1	13,911,458	1,846,479	82,175	11,982,804
	LO68_2	16,195,799	1,553,201	79,763	14,562,835
LO81	LO81_1	17,910,804	2,992,808	74,723	14,843,273
	LO81_2	15,646,123	5,115,030	61,672	10,469,421
Total		238,325,077	41,864,632	1,502,385	194,958,060
Average		14,895,317	2,616,540	93,899	12,184,879

**Fig. 2:**
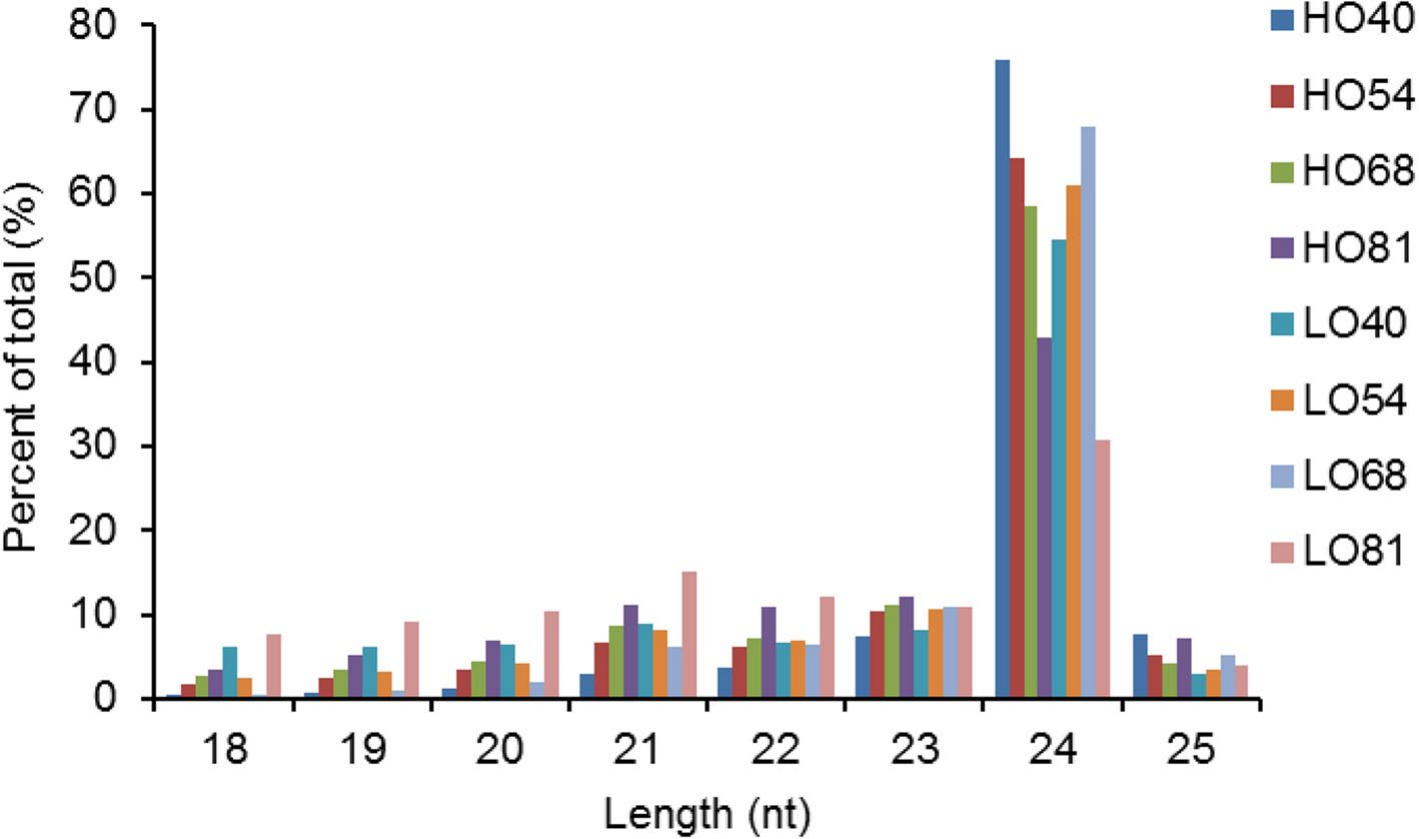
Length distribution of unique sRNAs in two yellowhorn lines at four developmental stages

Following a search against the Rfam and Repbase databases for sRNA sequences by the Bowtie software [30], an average of 3.22 and 7.17% clean reads in the HO and LO lines, respectively, were mapped to known non-coding sRNAs (rRNAs, tRNAs, snRNAs, snoRNAs, and other Rfam RNAs) and repeat sequences (Additional file 1: Table S1; Additional file 2: Table S2). Moreover, an average of 5.69 and 4.41% clean reads in the HO and LO lines, respectively, were mapped to NATs (Natural Antisense Transcripts) and phased siRNAs (phasiRNAs). Notably, about 5% of the clean reads per library, on average, were mapped to miRNAs in each line (Additional file 1: Table S1; Additional file 2: Table S2). The proportion of miRNAs declined at 81 daa compared with 40, 54, and 68 daa in the two lines, implying that a body of miRNAs were induced in the early and middle stages of seed development. The length distribution of unique sRNAs in both lines at four developmental stages were then summarized. Most sRNA reads ranged from 21 to 24 nt in length, leading to similar length distributions in both lines at the different developmental stages. Twenty-four nt sRNAs were the most abundant, accounting for 30.74% (LO81) to 75.95% (HO40) of the total (Fig. 2). Also common were 21, 22, and 23 nt sRNAs, which were more abundant than those of any other length besides 24 nt.

### Identification of known and novel miRNAs

**Fig. 3:**
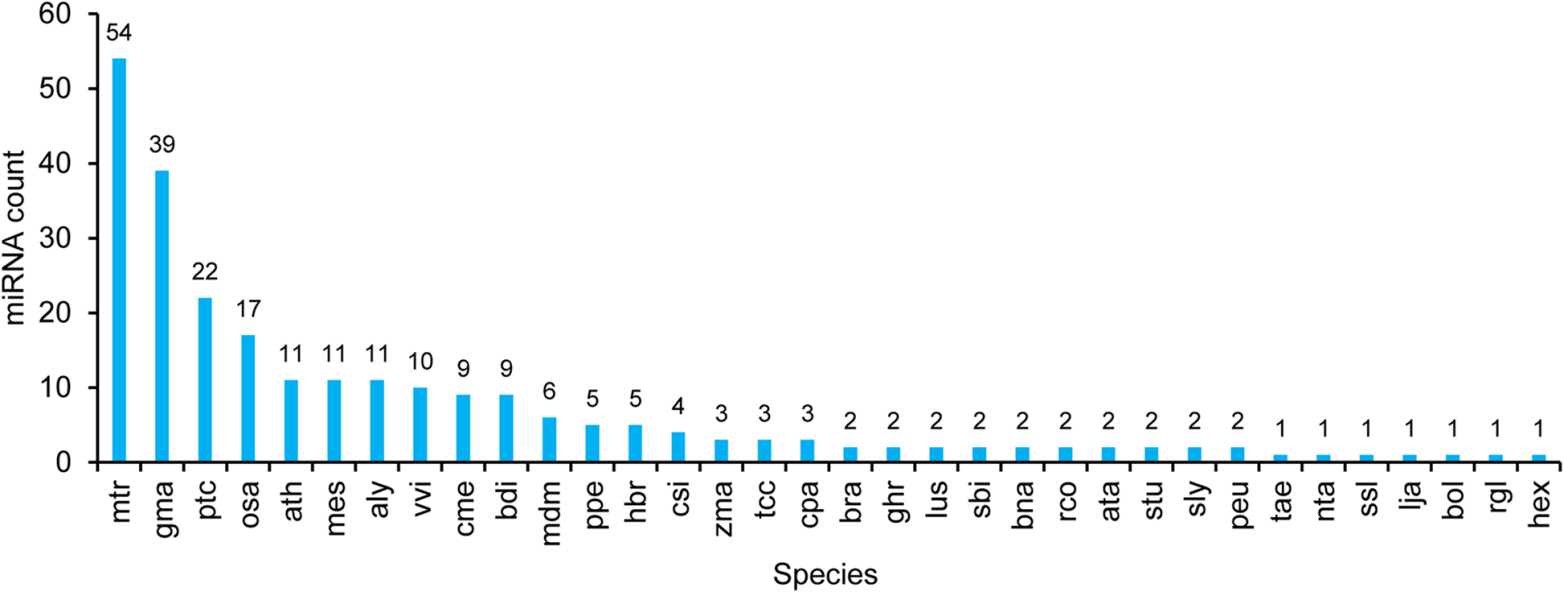
Species distribution of known miRNAs identified in yellowhorn

**Fig. 4:**
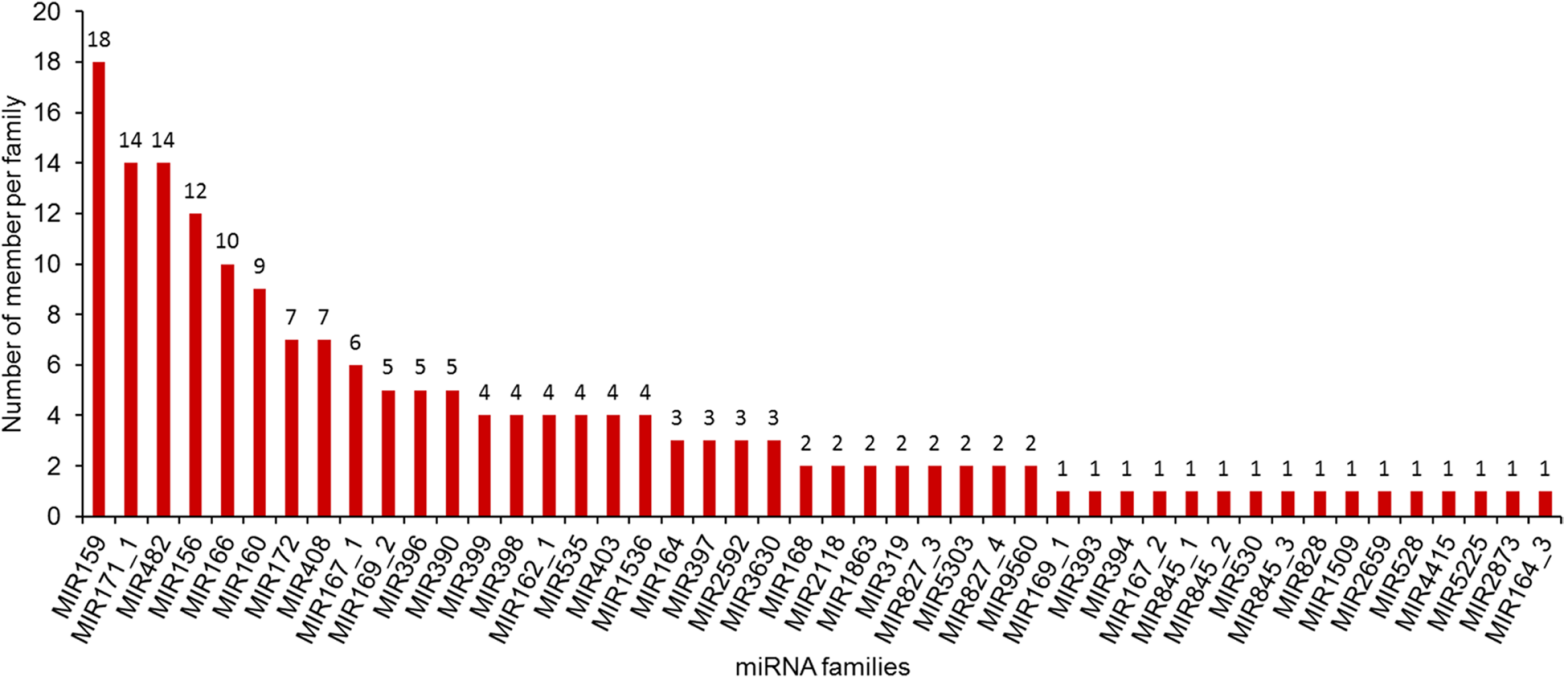
Number of identified miRNAs in each conserved miRNA family of yellowhorn

**Fig. 5:**
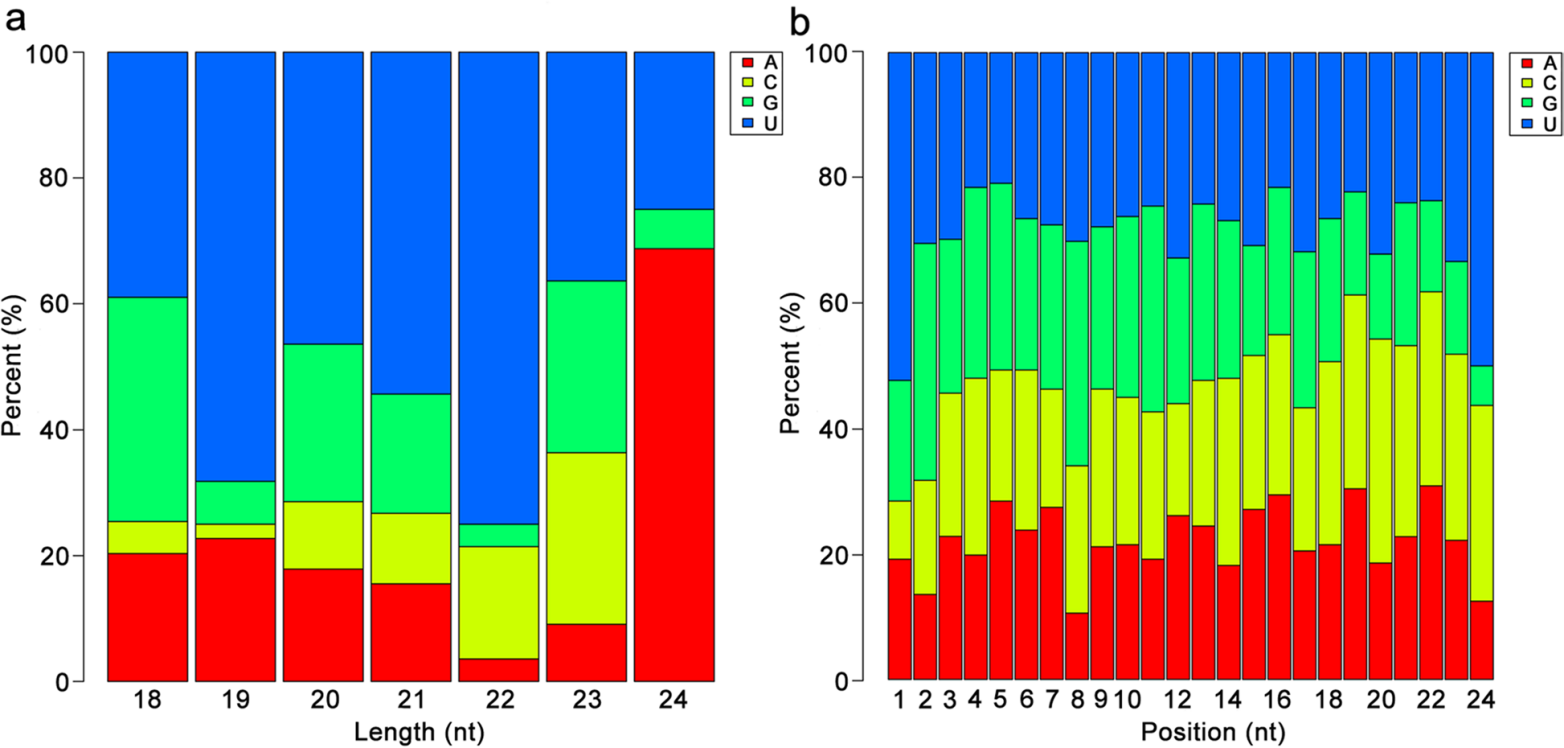
First nucleotide (**a**) and position nucleotide (**b**) biases of yellowhorn miRNA

A total of 249 known miRNAs were identified in the 16 libraries by analyzing the alignment results against the miRbase 21.0 database (Additional file 3: Table S3). Among the identified known miRNA sequences, 180 were identified to belong to 46 miRNA families. And the miRNA sequences were mapped to known miRNAs from 34 other plant species, with the highest number of miRNAs mapped to known mtr-miRNAs of *Medicago truncatula* (54, 21.67%), followed by gma-miRNAs of *Glycine max* (39, 15.66%), ptc-miRNAs of *Populus trichocarpa* (22, 8.84%), and osa-miRNAs of *Oryza sativa* (17, 6.83%) (Fig. 3; Additional file 4: Table S4). Among the identified miRNA families, the MIR159 family had the largest number of members (18), followed by the MIR171_1 and MIR482 families with 14 members, and MIR156 and MIR166, with 12 and 10 members, respectively (Fig. 4). The expression profiles of the known miRNAs showed that the normalized read counts of known miRNAs varied from 100,000 to less than 10 reads, exhibiting great variation, even within the same family (Additional file 3: Table S3). At the opposite end of the spectrum, about 49 miRNAs were identified that had fewer than 10 reads in all libraries. In addition, Fig. 5a shows the distribution of miRNA first nucleotide preferences. MiRNAs of 24 nt tended to begin with 5′-A, while 21–22 nt miRNAs tended to start with 5′-U. At the 5′ end, uridine was the most common nucleotide (> 52%) for all known miRNAs (Fig. 5b).

**Fig. 6:**
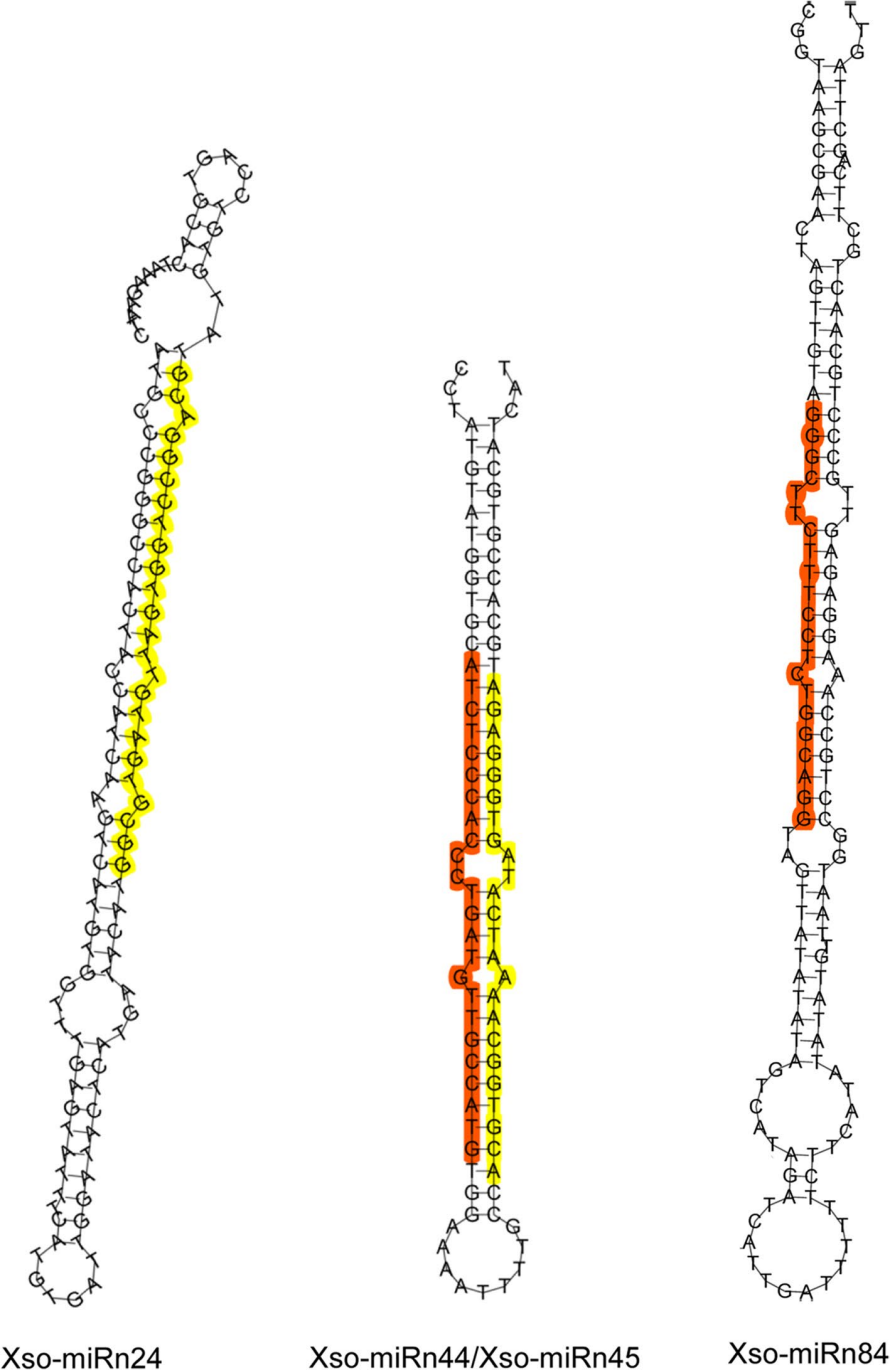
Secondary structures of the four most abundant novel miRNA precursors. Red (5p) and yellow (3p) indicate segments corresponding to the mature miRNAs

**Fig. 7:**
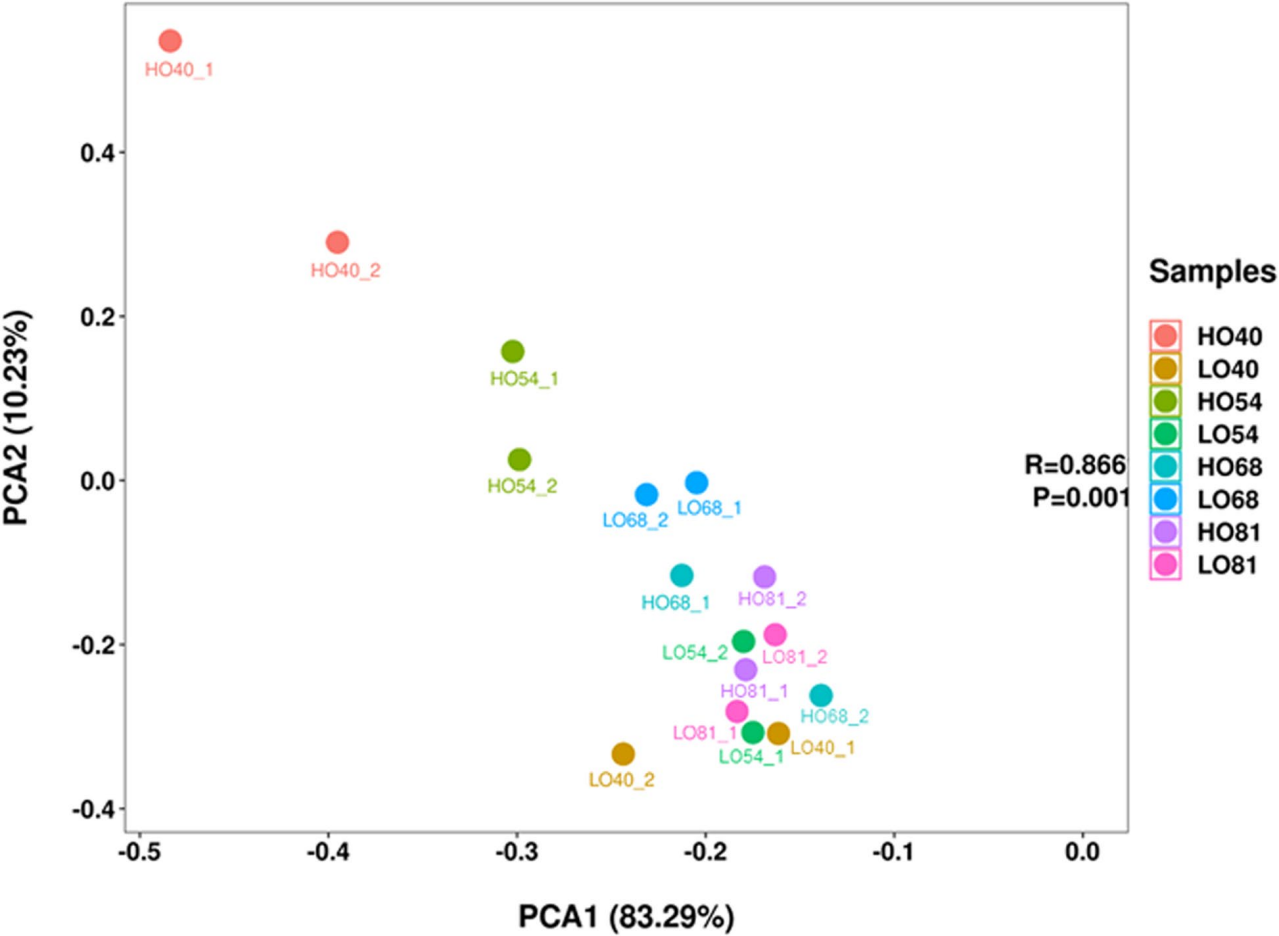
Principal Component Analysis (PCA) plot of each sample. The analyses were based on the normalized expression data for all miRNAs in 16 libraries. Percent of total variance captured by each principal component is shown in parentheses

In addition to the known miRNAs, the remaining unaligned sRNA sequences were mapped to the yellowhorn genome [31] and their hairpin structures were predicted to identify novel miRNAs in yellowhorn. We only considered sRNAs that exhibited the typical stem-loop structure. The novel miRNA sequences were named in the form of Xso-miRn-number. A total of 88 novel miRNAs between 18 nt to 25 nt in length were identified. Among them, 61.36% were 24 nt in length (Additional file 5: Table S5). The novel miRNA precursors ranged in length from 64 nt to 202 nt, with an average length of 140 nt. Compared to known miRNAs, most of the identified novel miRNAs had relatively low read counts (normalized). Most of the novel miRNAs had fewer than 10 reads, and only two novel miRNAs had more than 100 reads in all libraries (Additional file 5: Table S5). The minimum free energies of the hairpin structures of these miRNA precursors ranged from − 130.1 kcal·mol^− 1^ to − 26.9 kcal·mol^− 1^, with an average of − 59.6 kcal·mol^− 1^, similar to those of other plant miRNA precursors [32]. The secondary structures of the four most abundant novel miRNAs (Xso-miRn24, Xso-miRn44, Xso-miRn45, and Xso-miRn84) were predicted (Fig. 6), indicating they can form typical stem-loop hairpins, and their folding free energies were − 53.4, − 60.9, − 60.9, and − 60.8 kcal·mol^− 1^, respectively. Additionally, to evaluate the consistency of the biological replicates, Principal Components Analysis (PCA) was performed on the 16 samples using the expression profiles of all known and novel miRNAs. As shown in Fig. 7, every two replicates were relatively clustered together, indicating the reliability as well as operational stability of the experimental results.

### Differential expression of miRNAs in embryos during seed development

Pairwise comparison between different developmental stages in each line was conducted using the normalized expression levels of miRNAs to identify differentially expressed miRNAs (*P*-values < 0.05). In total, 53 known miRNAs and 11 novel miRNAs were found to be differentially expressed in pairwise comparisons (HO40 vs. HO54, HO54 vs. HO68, HO68 vs. HO81, LO40 vs. LO54, LO54 vs. LO68, and LO68 vs. LO81). Among them, only five known miRNAs (miR390, miR156i-3p_1, miR482b-3p_1, miR166a_1, and miR156e-3p_1) expressed at high levels (Additional file 6: Table S6; Additional file 7: Table S7).

In the HO line, 11 miRNAs were significantly differentially expressed between 40 and 54 daa (HO40 vs. HO54), including 5 and 6 miRNAs with upregulated and downregulated expression in HO54, respectively (Additional file 6: Table S6a). And 8 differentially expressed miRNAs were identified between 54 and 68 daa (HO54 vs. HO68), which included 3 upregulated miRNAs and 5 downregulated miRNAs in HO68 (Additional file 6: Table S6b). Furthermore, four miRNAs were upregulated and another four miRNAs were downregulated at the fully mature stage (81 daa) of the HO line compared with 68 daa (Additional file 6: Table S6c). In the LO line, there were 17 significantly differentially expressed miRNAs between 40 and 54 daa (LO40 vs. LO54). When compared to LO40, 6 and 11 miRNAs were upregulated and downregulated in LO54, respectively (Additional file 7: Table S7a). In the LO54 vs. LO68 comparison, 7 miRNAs were upregulated and 8 miRNAs were downregulated in LO68 (Additional file 7: Table S7b). Moreover, 20 miRNAs showed significant differential expression between 68 and 81 daa (LO68 vs. LO81), of which 10 miRNAs were upregulated and another 10 miRNAs were downregulated in LO81 (Additional file 7: Table S7c). Additionally, we found two differential miRNAs (upregulated miR156e-p3_1 and downregulated miR172b) in both lines at 40 vs. 54 daa and 54 vs. 68 daa, respectively.

### Differentially expressed miRNAs between the high-oil-content and low-oil-content lines

**Fig. 8:**
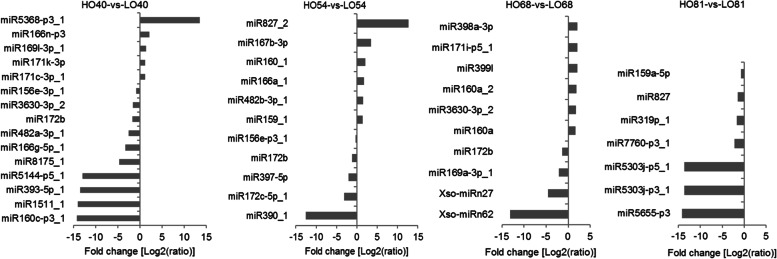
Differentially expressed miRNAs between the HO and LO lines during seed development

The differentially expressed miRNAs between the two lines were compared. The significantly differentially expressed miRNAs between two samples with a *P-*value < 0.05 are presented in Fig. 8. At 40 daa, 5 miRNAs were significantly upregulated and 10 miRNAs (e.g., miR156e-3p_1 and miR172b) were downregulated in the HO line compared with the LO line. At 54 daa, six miRNAs were upregulated in the HO line, while the other five miRNAs (miR172b, miR390_1, miR397-5p, miR172c-5p_1, and miR156e-p3_1) were downregulated in the HO line compared with the LO line. At 68 daa, six upregulated miRNAs (e.g., miR171i-p5_1) were found in the HO line, whereas the other four miRNAs (miR169a-3p_1, miR172b, Xso-miRn62, and Xso-miRn27) were preferentially expressed in the LO line. We also identified seven downregulated miRNAs (e.g., miR319p_1, miR5655-p3, and miR7760-p3_1) between fully mature embryos of the HO and LO lines. Interestingly, one co-expressed differential miRNA (miR172b) was detected that was downregulated at the early and middle stages of seed development (40, 54, and 68 daa) in the HO line relative to the LO line at the corresponding developmental stages. This indicated that this known miRNA might play an important role in the early and middle stages of yellowhorn seed development.

### Identification of differentially expressed miRNA target genes and gene enrichment analysis

Target mRNA is regulated by miRNA through translational repression or mRNA degradation. A total of 141 differentially expressed miRNAs (120 known and 21 novel miRNAs) were putatively targeted to 2654 genes to identify any correlation between the expression of differentially expressed miRNAs and mRNAs (Additional file 8: Table S8). Some miRNA target genes were annotated as transcription factor-coding genes, including *ARF2*, which was targeted by miR172b; *growth-regulating factor 5* (*GRF5*), which was targeted by miR171i-p5_1; *ethylene-responsive transcription factor 3* (*ERF3*), which was targeted by miR171k-5p_1; MADS domain protein *AGAMOUS-LIKE 61* (*AGL61*), which was targeted by miR7760-p3_1; and *WRKY transcription factor 41* (*WRKY41*), which was targeted by Xso-miRn80 (Additional file 9: Table S9). MiR7760-p3_1 was also predicted to target the *KAR* gene; miR319p_1 targets the *FAD2–2* gene; miR1536-p5_2 targets the *lysophosphatidyl acyltransferase 5* (*LPAT5*) gene; miR5647-p3_1 targets the *DGAT1* gene and miR7760-p5_1 was predicted to target the *Mediator subunit 15a* (*MED15A*) gene (Additional file 9: Table S9). These miRNA-target regulatory modules are reported here in yellowhorn for the first time.

**Fig. 9:**
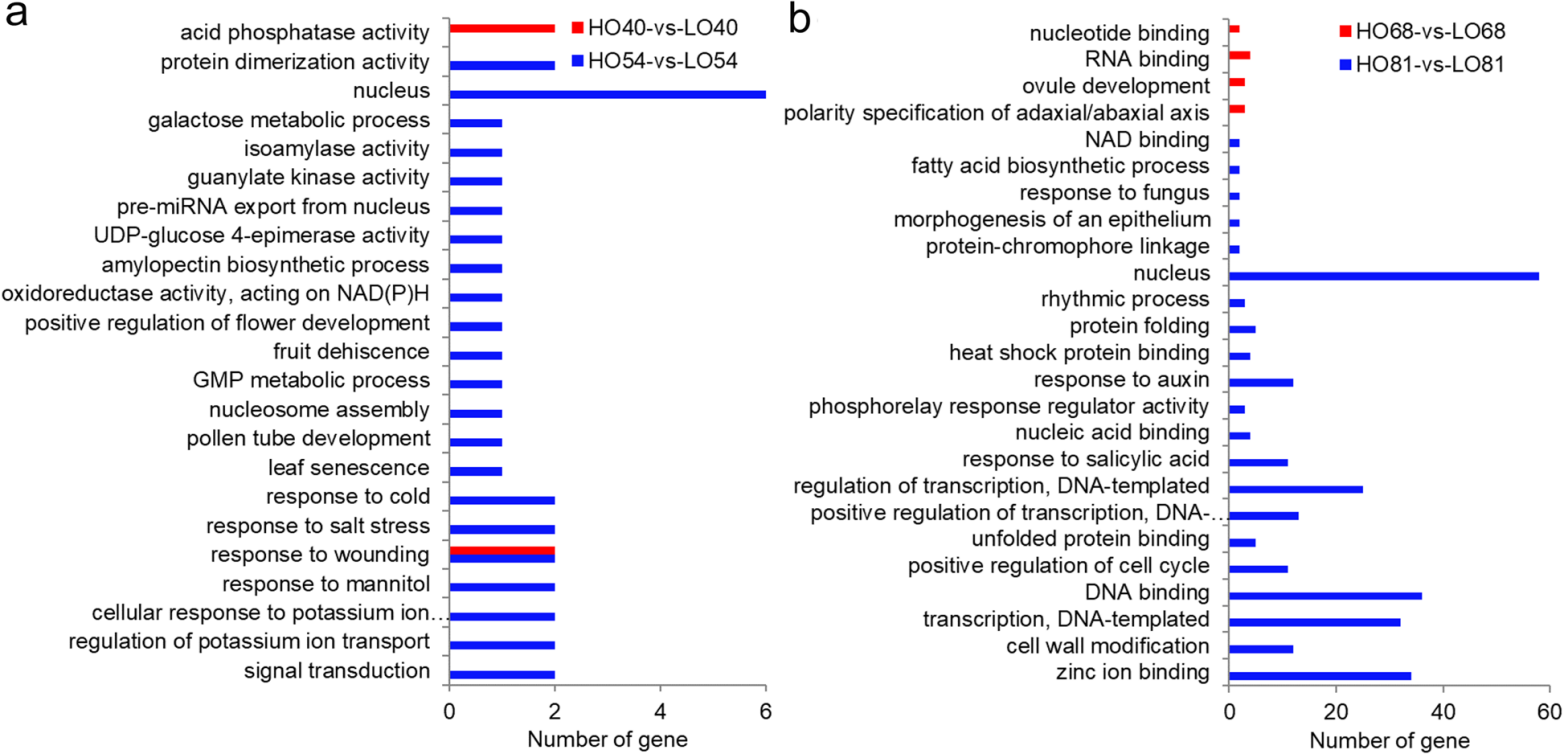
Gene Ontology categories and sub-categories of the differentially expressed miRNA targets between the following samples: **a** HO40 vs. LO40 and HO54 vs. LO54, and **b** HO68 vs. LO68 and HO81 vs. LO81

The differentially expressed miRNA targets between two lines were subjected to Gene Ontology (GO) enrichment analysis. One oil accumulation-related GO term (FA biosynthetic process (GO:0006633) was found to be significantly enriched by conducting GO annotation of these target genes. Notably, the most enriched GO terms were the nucleus, DNA binding, transcription, regulation of transcription, positive regulation of transcription, zinc ion binding, response to auxin, and cell wall modification (Fig. 9). The target genes of the differentially expressed miRNAs (miR7760-p3_1) in HO81 and LO81 were involved in the regulation of FA biosynthetic process, positive regulation of transcription, zinc ion binding, and NAD binding (Additional file 8: Table S8). The functions of the predicted target genes were diverse, suggesting that these differentially expressed miRNAs may play important roles during seed development and lipid biosynthesis in yellowhorn.

Kyoto Encyclopedia of Genes and Genomes (KEGG) pathway analysis of all differentially expressed miRNA targets showed that 268 target genes targeted by 52 of differentially expressed miRNAs mapped to 145 pathways (Additional file 10: Table S10), including those correlated with amino acid metabolism and lipid biosynthesis and metabolism, such as histidine metabolism, biosynthesis of unsaturated fatty acids, fatty acid biosynthesis, glycosphingolipid biosynthesis-ganglio series, glycerolipid metabolism, and fatty acid metabolism. Notably, the miRNAs miR319p_1, miR7760-p3_1, miR7760-p5_1, and miR5655-p3 were associated with biosynthesis of unsaturated fatty acids and fatty acid biosynthesis through targeting the *FAD2–2*, *KAR*, *short-chain type dehydrogenase/reductase* (*SDR1*), and *pentatricopeptide repeat-containing protein* (*PCMP-H42*) genes, respectively (Additional file 9: Table S9; Additional file 10: Table S10).

### Features and the evolutionary conservation of the target gene encoding MED15A

With respect to the target gene (cDNA sequence) encoding MED15A, which was designated as *XsMED15A*, it had 3467 nucleotide residues containing a complete 2463-bp open reading frame (ORF), which was predicted to code for 820 amino acids-polypeptide with a deduced molecular weight of 89.02 kDa and isoelectric point of 8.08 (Additional file 11: Fig. S1). XsMED15A amino acid sequence was submitted online and the results showed that it contained a coiled coil region and two internal repeats, without signal peptide. Multiple sequence alignments indicated that XsMED15A mature peptide was highly conserved with Mediator subunit 15a found in other plants. It shared 81% identity with the MED15A protein of *Pistacia vera* (XP_031251638.1), 80% with *Citrus clementina* (XP_024036133.1), 78% with *Juglans regia* (XP_035548095.1), 77% with *Malus domestica* (XP_028955279.1), 76% with *Gossypium hirsutum* (XP_040969330.1), and 74% with *G. max* (XP_003547623.1) (Additional file 12: Fig. S2).

### Quantitative real-time PCR validation of miRNA and corresponding target genes

**Fig. 10:**
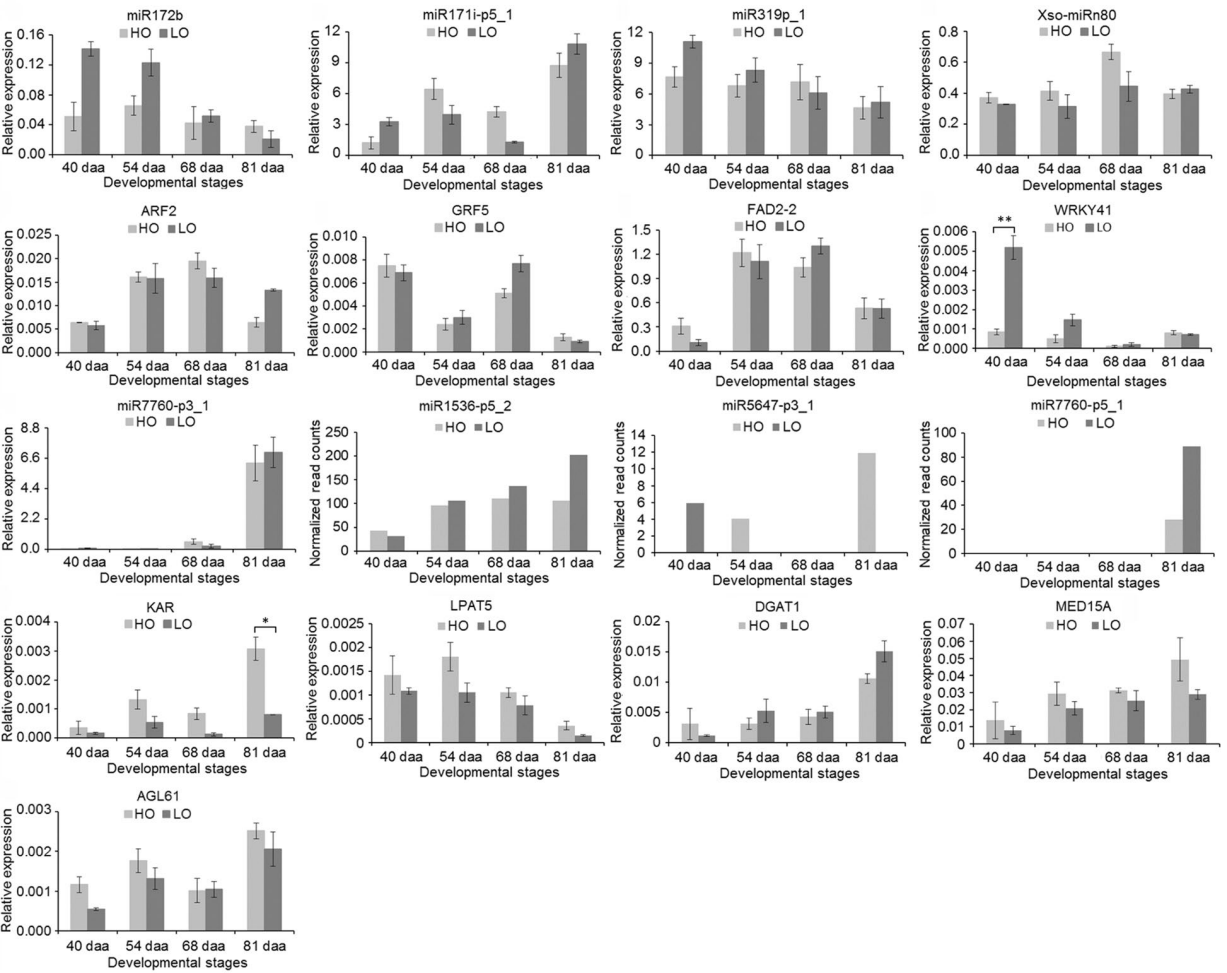
qRT-PCR validation of differentially expressed miRNAs and the target genes. The relative expression levels of five miRNAs (miR172b, miR171i-p5_1, miR7760-p3_1, miR319p_1, and Xso-miRn80) and all target genes were derived from the qRT-PCR analysis, and the normalized expression data of three miRNAs (miR1536-p5_2, miR5647-p3_1, and miR7760-p5_1) were derived from small RNA sequencing. The error bars indicate standard deviations for the three biological replicates. ** and * represent significant differences between the lines at the same developmental stage based on a Student’s T-test at *P* < 0.01 and *P* < 0.05, respectively

To validate the expression patterns of the miRNAs derived from the high-throughput sequencing, five miRNAs (miR172b, miR171i-p5_1, miR7760-p3_1, miR319p_1, and Xso-miRn80) and nine target genes (*ARF2*, *GRF5*, *AGL61*, *KAR*, *FAD2*–*2*, *WRKY41*, *LPAT5*, *DGAT1*, and *MED15A*) were selected and qRT-PCR analysis was performed. The resulting qRT-PCR data for the differentially expressed miRNAs were nearly consistent with the sequencing results, and the expression changes of the target genes showed an inverse correlation with differentially expressed miRNAs (Fig. 10). MiR7760-p3_1, however, showed relatively low expression in both lines at 40–68 daa compared with 81 daa, and showed exclusive expression at 81 daa in the sequencing data.

## Discussion

### Complex sRNA populations involved in seed development and lipid biosynthesis of yellowhorn

Yellowhorn is an endemic and economically important oil-rich tree in northern China that can withstand cold and drought conditions [13]. The oil production of oil plant seeds is mainly determined by the seed size, seed weight, and embryo oil content. However, the complex regulatory mechanisms of seed development and oil biosynthesis affecting oil production in yellowhorn are still poorly understood. In this study, the dynamic growth patterns (size and weight) and the embryo oil content of developing seeds in HO and LO yellowhorn lines were analyzed, and faster growth and higher oil accumulation in developing seeds were detected at 40–54 daa and 40–68 daa, respectively (Fig. 1). Furthermore, the HO line had a higher embryo oil content, higher hundred-grain weight, and smaller seeds than the LO line (Fig. 1). Though these HO and LO lines shared similar genetic backgrounds, they exhibited large phenotypic differences, which are of interest in terms of attempts to understand their underlying molecular mechanisms.

MiRNAs as systemic regulators are involved in plant seed development and lipid metabolism [17, 25, 33]. Although some functional genes responsible for lipid biosynthesis in developing yellowhorn seeds were cloned and identified in previous studies [9, 10, 12], the role of miRNAs in seed developmental processes, for example, seed size, and lipid biosynthesis, remains unknown. In this study, we constructed sRNA populations of the HO and LO lines at four seed developmental stages. The 16 sRNA libraries had abundant high-quality data. From the yellowhorn embryo sRNA library, 249 known miRNAs belonging to 46 families were identified, and conserved across 34 plant species, including notable conservation with *M. truncatula*, *G. max*, and *P. trichocarpa*; this suggested a close relationship between these plant species and yellowhorn, nearly in accordance with the report by Wang et al [29]. Some known miRNAs from 9 miRNA families (e.g., MIR156, MIR159, MIR168, MIR166, and MIR482) demonstrated high expression levels in all libraries, validating their broad evolutionary conservation. In addition, 88 novel miRNAs were identified, most of which showed much lower expression levels than the known miRNAs. Only two novel miRNAs (Xso-miRn42 and Xso-miRn44) exhibited relatively higher expression levels (normalized read counts > 100) in all libraries.. These findings suggested that a complex, diverse array of sRNAs were involved in yellowhorn seed development and lipid biosynthesis.

### MiRNAs and their target genes associated with the seed development

Auxin is implicated in various physiological and developmental processes in plants [34]. Many investigations indicated that auxin response factors regulated plant seed development by controlling auxin responses [33]. For example, abnormal embryo symmetry (tri- or quadrilateral instead of bilateral cotyledons) defects occurred in plants that expressed miR160-resistant ARF17 [35]. ARF2 is linked to seed size in plants [36]. In *Arabidopsis*, the *megaintegumenta* (*mnt*) mutant, which has a lesion in the *ARF2* gene, was found to produce larger seeds than wild-type *Arabidopsis* [37]. In the present study, miR172b was predicted to target the *ARF2* gene. The *ARF2* gene showed slightly higher expression in HO than in LO at 40–68 daa, with an exception occurring at 81 daa (Fig. 10). In contrast, miR172b was found to be downregulated during seed development in HO compared to LO in the early to middle stages (Figs. 8 and 10). These data combined with the finding that the HO line exhibited smaller seeds compared to the LO line (Fig. 1c–e) suggested that miR172b might positively regulate seed size in yellowhorn through the suppression of the *ARF2* gene. Previous studies showed that the miR172 family positively affected seed weight through the suppression of APETALA2-like transcription factors in *Arabidopsis* [38, 39]; thus, we presume that the members of the miR172 family have diverse biological functions in the seed development of plants.

The growth-regulating factor family is composed of plant-specific transcription factors that play various roles in growth and development, such as in seed formation [40, 41] and leaf development [42]. In *A. thaliana*, AtGRF5 participated in the positive control of cell proliferation during leaf development [43]. Owing to an increased cell number, *AtGRF5* overexpression lines had larger leaf areas, while the *atgrf5* single-gene mutant had narrower leaves compared to the wild type due to a decreased cell number [43]. Furthermore, *GRF5*-overexpressing lines had improved photosynthetic performance, increased chloroplast number, and higher tolerance to nitrogen depletion compared with wild-type plants [44]. A large body of research has determined that miR396 directly regulates the expression of some *GRFs* at the post-transcription level [16, 45]. However, *AtGRF5* has no miR396 target site and is therefore not regulated by this miRNA in *A. thaliana* [46, 47]. Notably, miR171i-p5_1 targets the *GRF5* gene in yellowhorn seeds. MiR171i-p5_1 exhibited decreased expression from 54 to 68 daa, followed by a sharp increase at 81 daa in the two lines, while *GRF5*, its target gene, exhibited a trend opposite to that of miRNA (Fig. 10). Additionally, the expression level of *GRF5* was lower in HO than in LO at 54 and 68 daa, whereas miR171i-p5_1 was more highly expressed in HO compared to LO at the corresponding developmental stages and was particularly significantly upregulated at 68 daa (Figs. 8 and 10). These results suggested that the *GRF5* gene is negatively regulated by miR171i-p5_1 in yellowhorn, and smaller seeds in the HO line may be attributed to a negative regulatory role of miR171i-p5_1.

MADS-box genes are critical in plant development, especially in gamete and seed development. The MADS domain protein AGL62 regulates the timing of endosperm cellularization [48]. Recently, research has shown that OsMADS87 plays a role in endosperm cellularization initiation and in the regulation of the final seed formation in rice [49]. *AGL61* was also found to be expressed in the central and endosperm cells. In the *agl61* mutant central cell development was impaired, resulting in a maternal-lethal phenotype [50]. In this study, we found that the *AGL61* gene, targeted by miR7760-p3_1, showed higher expression in HO than in LO at 40, 54, and 81 daa (Fig. 10). As expected, miR7760-p3_1 showed lower expression in HO compared to LO at 40, 54, and 81 daa and was particularly significantly downregulated in HO at 81 daa (Figs. 8 and 10), indicating that miR7760-p3_1 mainly functions in the mature stage of seed development by negatively regulating the expression of *AGL61*. In a previous study, it was shown that after pollination, the yellowhorn embryo sac gradually accumulated a high liquid content, including free nuclear endosperm, a small amount of cellular endosperm, and other soluble material [51]. In addition, when the ovule develops into a mature seed, the endosperm is completely absorbed by the embryo. Therefore, the increase of seed weight in the HO line compared to LO may be the result of an increase in seed filling during the maturation phase due to greater absorption of the endosperm caused by higher *AGL61* expression. Further work is clearly required to completely understand the roles and involvement of AGL61 in yellowhorn seed development.

### MiRNAs and their targets are related to yellowhorn lipid biosynthesis

The sequencing of oil crops has yielded many miRNAs associated with lipid metabolism-related genes in recent years. For example, 97, 40, 30, and 4 miRNAs targeting 89, 15, 133, and 4 lipid biosynthesis genes were reported for soybean [52], *B. napus* [24], *C. sativa* [21], and peanut [22], respectively. Here, we detected five differentially expressed miRNAs targeting five candidate genes regulating lipid biosynthesis functions during seed development. Among these miRNAs, miR7760-p3_1 regulates *KAR*, which is directly involved in fatty acid biosynthesis [11, 53], as well as *AGL61*, which is involved in seed development. In contrast to miR7760-p3_1, the *KAR* gene, which encodes 3-ketoacyl-ACP reductase and catalyzes the formation of C16:0-ACP or C18:0-ACP as a component of the FA synthase (FAS) complex [11, 53], exhibited a higher expression level in HO than in LO during seed development (Fig. 10). The *KAR* gene showed increased expression from 40 to 54 daa, reduced expression at 68 daa, and finally increased expression at the mature stage in the two lines. These results were in accordance with the changes in the relative percentages of palmitic acid (C16:0) and stearic acid (C18:0) of total oils during seed development [54]. Similarly, miR319p_1 was significantly downregulated in HO compared with LO at 81 daa (Fig. 8), and expression of its target gene *FAD2–2*, which desaturates oleic acid (C18:1) to form linoleic acid (C18:2) [11], was slightly increased in HO at 81 daa (Fig. 10). The expression level of the *FAD2–2* gene rapidly increased from 40 to 54/68 daa in the two lines and then decreased at the mature stage (Fig. 10), which correlated well with the changes in linoleic acid and the total FA content in developing yellowhorn seeds [8]. Extensive research suggests that FA biosynthesis in higher plants may be an important regulatory step in oil accumulation [55, 56]. The enhanced expression of *KAR* and *FAD2–2* genes in the HO line could thereby promote elevated oil accumulation by contributing to an increased FA supply in HO yellowhorn, revealing that miR7760-p3_1 and miR319p_1 may participate in the regulation of lipid biosynthesis by regulating FA biosynthesis genes *KAR* and *FAD2–2*, respectively.

Activated FAs in the form of acyl-CoA are sequentially incorporated into glycerol-3-phosphate to produce triacylglycerols (TAGs) in the TAG synthesis process via the Kennedy pathway. These TAGs are then catalyzed by a series of enzymes, including LPAT and DGAT [57]. In our study, the expression level of *LPAT5*, targeted by miR1536-p5_2, was higher in HO than in LO during seed development (Fig. 10), while miR1536-p5_2 showed lower expression in HO compared to LO at 54, 68, and 81 daa (Fig. 10; Additional file 3: Table S3). Combined with the findings that the HO line showed higher oil content than the LO line during seed development, it is therefore likely that miR1536-p5_2 mainly played a key regulatory role in lipid biosynthesis in the middle and mature stages of seed development by negatively regulating the TAG biosynthesis gene *LPAT5*. A rate-limiting reaction in TAG biosynthesis is catalyzed by the DGAT enzyme, and the *DGAT1* and *DGAT2* genes control the progression of this reaction [10]. Overexpression of *AtDGAT1* in seeds increased the seed oil content by up to 8.3% in transgenic *B. juncea* compared to that in wild-type plants [58]. In yellowhorn, the *DGAT1* gene, targeted by miR5647-p3_1, was more highly expressed in the HO line compared to the LO line at 40 daa (Fig. 10), which was in accordance with the difference in the embryo oil content between the two lines. Unlike the *DGAT1* gene, miR5647-p3_1 expression was only lower in the HO line at 40 daa (Fig. 10; Additional file 3: Table S3). Thus, it is speculated that miR5647-p3_1 affected primarily seed oil accumulation at the early stage of yellowhorn seed development through the suppression of the *DGAT1* gene. Although the expression patterns of miR5647-p3_1 cannot fully explain the difference in the embryo oil content in the two lines, the possibility of regulation of *DGAT1* by miR5647-p3_1 cannot be ruled out at all seed developmental stages. Further molecular genetics studies should be used to determine the role of miR5647-p3_1 in the lipid biosynthesis process.

The Mediator complex plays an essential role in transcriptional regulation in eukaryotes by connecting DNA-binding transcription factors to the RNA polymerase II transcription machinery. It is a large complex comprising 25 to 30 different protein subunits [59, 60]. MED15 is a subunit of the tail module of the Mediator complex. MED15 includes a kinase-inducible domain-interacting domain at its N-terminal region that mediates protein–protein interactions through a hydrophobic pocket [61]. Several previous studies suggested that MED15 plays an important regulatory role in diverse biological processes, including lipid metabolism, seed development, and defense signaling pathways in many plants [62, 63]. A recent study found that the *Arabidopsis* MED15 subunit interacts directly with the WRINKLED1 transcription factor during embryogenesis to mediate the activation of downstream lipid-related genes. The overexpression of *MED15* increased the transcription of glycolysis-related and FA biosynthetic genes, resulting in increased FA content in mature seeds [64]. In yellowhorn, the *MED15A* gene is targeted by miR7760-p5_1. Interestingly, miR7760-p5_1 showed decreased expression in the HO line compared with the LO line at the mature stage (Fig. 10; Additional file 3: Table S3), which led to higher expression of the *MED15A* gene in the HO line (Fig. 10). These changes might improve oil accumulation in the HO line by activating downstream lipid-related genes and further increasing FA biosynthesis. Because a few varieties of FAs and total oil accumulated relatively rapidly between 40 and 68 daa and peaked at the mature stage [8–10], this study focused mainly on activities between 40 and 81 daa, the key period for oil accumulation. Expanding the scope of this work to focus on activities throughout seed development would provide a clearer picture of seed development and the initial stage of oil accumulation.

## Conclusions

In this work, embryos from four seed development stages of two yellowhorn lines (HO and LO) were used to construct separate small RNA libraries, including two replicates for each sample. A total of 249 known miRNAs belonging to 46 families and 88 novel miRNAs were identified in developing yellowhorn embryos as a result. We screened the differentially expressed miRNAs to obtain miRNAs involved in lipid biosynthesis and seed development regulation by comparing different yellowhorn lines and different developmental stages. The results identified many miRNA-target regulatory modules that have potential functions in regulating seed size, seed weight, and lipid biosynthesis in yellowhorn, including miR172b-*ARF2*, miR7760-p3_1-*AGL61*, miR319p_1-*FAD2–2*, miR5647-p3_1-*DGAT1*, and miR7760-p5_1-*MED15A*. These data will be valuable for dissecting the post-transcriptional and transcriptional regulation of seed development and lipid biosynthesis, as well as improving yellowhorn in northern China.

## Methods

### Plant materials and seed phenotype

Yellowhorn blooms early in May and its fruit ripens in late July. Two yellowhorn lines, NM1203 and NM1003, were grown at the Baxiantong Forest Farm located in Baxiantong Town, Naiman County, Tongliao City, Inner Mongolia Autonomous Region, China (121.04^°^ E, 43.21^°^ N). The two lines were bred by Dalian Minzu University and established by massal selection of systematic breeding of natural populations. Based on the average yearly embryo oil content, as assessed by bunch analysis over a 3-year period, two yellowhorn lines (NM1203 and NM1003) with similar genetic backgrounds were selected as the HO line and LO line, respectively. The permission was obtained from Dalian Minzu University for the collection of yellowhorn fruits. Fruits were collected at random from different plants of both lines at 40, 54, 68, and 81 daa in 2016, which were the same as those for published mRNA-seq data [12]. After collection, the seeds were separated from fruits at the four developmental stages by dissection. The phenotype characters of the seeds were evaluated. These included the hundred-grain weight, transverse diameter, longitudinal diameter, and lateral diameter. The analyses were repeated in triplicate. Liquid nitrogen was used to freeze the fresh embryo tissues and they were stored at − 80 °C for subsequent use.

### Oil content analysis

The seeds harvested at different times were dried to a constant weight at 80 °C and then ground using a ball mill. The oil was extracted from the embryos at 40, 54, 68, and 81 daa as previously described [65]. The experiments were repeated in triplicate.

### RNA isolation, small RNA library construction, and sequencing

Total RNA was extracted using a Trizol reagent (Invitrogen, Carlsbad, CA, USA) from embryo samples of both lines following the manufacturer’s protocol. A NanoDrop ND-2000 spectrophotometer (Thermo Fischer Scientific, Waltham, MA, USA) was used to assess the purity and quality of RNA. RNA integrity was assessed using the RNA Nano 6000 LabChip Kit of the Agilent Bioanalyzer 2100 system (Agilent Technologies, Santa Clara, CA, USA). sRNA libraries were prepared using the total RNA samples from embryos at each developmental stage from both lines following the protocol of the TruSeq Small RNA Sample Prep Kit (Illumina, San Diego, CA, USA). An Illumina HiSeq2500 Genome analyzer (Illumina) was then used to evaluate and sequence the purified library products at LC Sciences (Hangzhou, China), generating 50-bp single-end reads. Two independent replicates were used for each seed developmental stage from the LO and HO lines.

### Primary analysis of the sequencing data sets

After sequencing, the Illumina pipeline filter (Solexa 0.3) was used to process the raw reads to remove the adaptor dimers, junk reads, and low-complexity sequences, as well as those shorter than 18 nt and longer than 25 nt. The resulting clean reads were then mapped onto the Rfam (version 11.0) database (http://rfam.xfam.org/) and Repbase (http://www.girinst.org/) to remove rRNAs, tRNAs, snRNAs, snoRNAs, other Rfam RNAs, and repeat sequences using the Bowtie software (version 1.0.0) [30] in an in-house script ACGT101-miR (version 4.2) (LC Sciences, Houston, Texas, USA) [66], with seed length set to 16 and allowing one mismatch. The remaining sRNA reads after screening were retained for subsequent analysis.

### Identification of phasiRNAs and natural antisense siRNAs

The remaining sRNA reads were mapped to whole genome sequences of yellowhorn [31] using the Bowtie software [30], with seed length set to 16 and allowing one mismatch, to analyze their distribution relative to the reference sequence. The mapped sRNA reads were used to search for phasiRNAs and natural antisense siRNAs (NAT-siRNAs). The sRNA annotation process was conducted as follows: (1) The mapped sRNA reads were used to predict phasiRNAs using the PhaseTank software (version 1.0) [67] with the parameters --island 105 --phasiRNA_abun 50. (2) In the process of NAT-siRNAs identification, NAT gene prediction of the yellowhorn genome was first performed using PlantNATsDB platform [68]. The mapped sRNA reads were further aligned to NAT sequences to identify the sRNA reads originating from NATs using the Bowtie software with the parameters -v 1 -k 1. These sRNA reads originating from NATs were then used to predict clusters (NAT-siRNAs) using the ShortStack software [69] with default parameters.

### Identification and prediction of known and novel miRNAs

Known miRNAs were identified by aligning the remaining sRNA reads against the miRBase (version 21.0) database (http://www.mirbase.org) using the Bowtie software [30]. In the alignment, seed length was set to 16 and one mismatch inside the sequence was allowed. The mapped miRNA precursors (hairpins) in the miRBase were further aligned against whole genome sequences of yellowhorn [31] to determine their genomic locations using Megablast software in the in-house script ACGT101-miR, with identity percentage > 90%. Here, known miRNAs were sRNAs that could be aligned to any mature miRNA or miRNA precursor (pre-miRNA) from the plants in miRBase, allowing for up to one mismatch. New 5p- or 3p-derived miRNAs were the sRNAs that matched the other arm of known plant pre-miRNA hairpins opposite the annotated mature miRNA-containing arm. The newly discovered 5p/3p sequence was annotated as p5/p3, which differs from reported sequences.

After known miRNA prediction, the Bowtie software [30] was used to align remaining unaligned sRNA reads to whole genome sequences of yellowhorn [31], with seed length set to 16 and allowing one mismatch, to identify potentially novel miRNAs. The sRNA flanking genome sequences (120 nt upstream and 120 nt downstream) were extracted and used with RNAfold software (http://rna.tbi.univie.ac.at/cgi-bin/RNAfold.cgi) to predict the RNA secondary structure. The parameters were as follows: (1) number of nucleotides in one bulge of the stem (<= 12); (2) number of base pairs in the stem region of the predicted hairpin (> = 16); (3) cutoff of free energy (kcal/mol < = − 15); (4) length of the hairpin (upward and downward stems + terminal loop > = 50); (5) length of hairpin loop (<= 200); (6) number of nucleotides in one bulge in the mature region (<= 4); (7) number of biased errors in one bulge in the mature region (<= 2); (8) number of biased bulges in the mature region (<= 2); (9) number of errors in the mature region (<= 4); (10) number of base pairs in the mature region of the predicted hairpin (> = 12); and (11) mature region percentage in the stem (> = 80). After alignment analysis, the miRNAs that met the above criteria were considered novel miRNAs.

### Differential expression analysis of miRNAs

The number of clean reads originating from each miRNA represents the expression abundance or level of the corresponding miRNA in small RNA deep sequencing. Normalized read counts were calculated for miRNA differential expression analysis according to the following procedures: (1) Find a common set of sequences among all samples. (2) Construct a reference data set. Each data in the reference set is the copy number median value of a corresponding common sequence of all samples. (3) Perform 2-based logarithm transformation on copy numbers (*log*_2_(*copy*#)) of all samples and reference data set. (4) Calculate the *log*_2_(*copy*#) difference (∆*log*_2_(*copy*#)) between individual sample and the reference data set. (5) Form a subset of sequences by selecting |∆*log*_2_(*copy*#)| < 2, which means less than (2^2^ =) 4 fold change from the reference set. (6) Perform linear regressions between individual samples and the reference set on the subset sequences to derive linear equations y = *a*_*i*_*x* + *b*_*i*_, where *a*_*i*_ and *b*_*i*_ are the slop and interception, respectively, of the derived line, x is *log*_2_(*copy*#) of the reference set, and y is the expected *log*_2_(*copy*#) of sample i on a corresponding sequence. (7) Calculate the mid value *x*_*mid*_ = $\frac{\max \left(\mathrm{x}\right)-\min \left(\mathrm{x}\right)}{2}$ of the reference set. Calculate the corresponding expected *log*_2_(*copy*#) of sample i, *y*_*i*,*mid*_ = *a*_*i*_*x*_*mid*_ + *b*_*i*_. Let *y*_*r*,mid_ = *x*_*mid*_, let ∆*y*_*i*_ = *y*_*r*,mid_-*y*_*i*,mid_, which is the logarithmic correction factor of sample i. We then derive the arithmetic correction factor *f*_*i*_ = 2^Δ*yi*^ sample i. (8) Correct copy numbers of individual samples by multiplying corresponding arithmetic correction factor fi to original copy numbers. A Student’s T-test was used to identify differentially expressed miRNAs between two samples based on normalized read counts. An analysis of variance (ANOVA) was used to identify differentially expressed miRNAs among samples at the four development stages in each line. The miRNAs with *P*-values < 0.05 were considered differentially expressed.

### Target gene predication and enrichment analysis

The target genes of differentially expressed miRNAs were identified by aligning mature miRNA sequences with published mRNA-seq sequences [12] using TargetFinder (https://github.com/carringtonlab/TargetFinder) following the procedures described in previous studies [70, 71]. To study the biological significance of the differentially expressed miRNA targets, GO enrichment analyses were conducted as previously described [12]. GO terms with a *P*-value < 0.05 were considered significantly enriched. To determine the biochemical metabolic pathways and signal transduction pathways involved by the differentially expressed miRNA targets, KEGG enrichment analyses were performed as those used for GO analysis. Pathways with a *P*-value < 0.05 were considered significantly enriched by differentially expressed miRNA targets.

### Sequence analysis of the target gene

The target gene encoding Mediator subunit 15a (XsMED15A) was analyzed using the BLAST program and ORF Finder tool at NCBI (http://www.ncbi.nlm.nih.gov/) for the homology study and ORF prediction. The analysis of deduced polypeptides was performed with ExPASy tool (https://www.expasy.org/), which has hyperlinks to different prediction programs, including the ProtParam program for physicochemical properties prediction. Domains, motifs, and other features were identified using the online program SMART (http://smart.embl-heidelberg.de/). The DNAMAN program was used for multiple sequence alignment.

### Expression analysis of miRNAs and predicted target genes using qRT-PCR

The total RNA was isolated from the embryo samples from both lines as described under sRNA library preparation. One microgram of total RNA was used for first-strand cDNA synthesis using PrimeScript RT reagent Kit with gDNA Eraser (TaKaRa, Dalian, China) following the manufacturer’s protocol. For miRNA expression analysis, the Mir-X miRNA First-Strand Synthesis Kit (Takara) was used to perform first-strand cDNA synthesis following the manufacturer’s instructions. qRT-PCR was performed using TB Green Premix Ex Taq II (Tli RNaseH Plus) (TaKaRa) on an Applied Biosystems 7500 Real-Time PCR System (Applied Biosystems, Foster City, CA, USA) with the following cycling conditions: 95 °C for 30 s, followed by 40 cycles of 95 °C for 5 s and 60 °C for 30 s. The miRNA expression-level analysis used yellowhorn 5.8S rRNA as an internal reference, while target expression-level analysis used the *β*-*actin* gene as an internal control. The 2^-Δ^CT method was used to calculate the relative expression levels of each miRNA and their targets [72]. The primer sequences used to validate miRNA and target gene expression are listed in Additional file 13: Table S11 and Additional file 14: Table S12, respectively. Three biological replicates were conducted to obtain the data.

## Supplementary Information

**Additional file 1: Table S1.** Distribution of small RNAs among different categories in HO yellowhorn.

**Additional file 2: Table S2.** Distribution of small RNAs among different categories in LO yellowhorn.

**Additional file 3: Table S3.** The known miRNAs identified in yellowhorn.

**Additional file 4: Table S4.** Species and abbreviations of the known miRNAs of yellowhorn.

**Additional file 5: Table S5.** The novel miRNAs identified in yellowhorn.

**Additional file 6: Table S6.** (a) Differentially expressed miRNAs between HO40 and HO54. (b) Differentially expressed miRNAs between HO54 and HO68. (c) Differentially expressed miRNAs between HO68 and HO81.

**Additional file 7: Table S7.** (a) Differentially expressed miRNAs between LO40 and LO54. (b) Differentially expressed miRNAs between LO54 and LO68. (c) Differentially expressed miRNAs between LO68 and LO81.

**Additional file 8: Table S8.** Targets prediction of all differentially expressed miRNAs.

**Additional file 9: Table S9.** Identified candidate targets for partial known and novel miRNAs.

**Additional file 10: Table S10.** Enrichment analysis of KEGG pathways of miRNAs and their target genes of yellowhorn.

**Additional file 11: Figure S1.** Nucleotide and deduced amino acid sequence of cDNA encoding Mediator subunit 15a (XsMED15A). The start codon is marked in red color font. An asterick represents the stop codon.

**Additional file 12: Figure S2.** Multiple sequence alignment of Mediator subunit 15a from different species. The MED15A protein sequences were chosen from different species and aligned by DNAMAN. The two internal repeats within XsMED15A are shown in black boxes. The predicted coiled coil region is underlined. GenBank accession numbers of MED15A protein sequences used for the comparison are showed as follows: *Xanthoceras sorbifolium* (BankIt2507240 XsMED15A OK432284), *Pistacia vera* (XP_031251638.1), *Citrus clementina* (XP_024036133.1), *Juglans regia* (XP_035548095.1), *Malus domestica* (XP_028955279.1), *Gossypium hirsutum* (XP_040969330.1), *Arachis hypogaea* (XP_025617476.1), *Medicago truncatula* (XP_024636817.1), *Brassica rapa* (XP_033134666.1), and *Glycine max* (XP_003547623.1).

**Additional file 13: Table S11.** Primers for qRT-PCR analysis of differentially expressed miRNAs.

**Additional file 14: Table S12.** Primers for qRT-PCR analysis of target genes of differentially expressed miRNAs.

## Data Availability

The datasets generated and analysed during the current study are available in the NCBI Sequence Read Archive (SRA) database under Bioproject PRJNA493982 (https://www.ncbi.nlm.nih.gov/bioproject/?term=PRJNA493982).

## Abbreviations

miRNA: MicroRNA
sRNA: Small RNA
HO: High-oil-content
LO: Low-oil-content
daa: Days after anthesis
NAT-siRNAs: Natural antisense siRNAs
phasiRNAs: Phased siRNAs
FAs: Fatty acids
DGAT: Diacylglycerol acyltransferase
KAR: 3-oxoacyl-ACP reductase
FAD2: Omega-6 fatty acid desaturase 2
FAE1: Fatty acid elongase 1
FATB: Fatty acyl-ACP thioesterase B
ARF: Auxin response factor
GRF5: Growth-regulating factor 5
ERF3: Ethylene-responsive transcription factor 3
AGL61: AGAMOUS-LIKE 61
WRKY41: WRKY transcription factor 41
LPAT5: Lysophosphatidyl acyltransferase 5
MED15A: Mediator subunit 15a
SDR1: Short-chain type dehydrogenase/reductase
PCMP-H42: Pentatricopeptide repeat-containing protein
FAS: FA synthase
TAG: Triacylglycerol
GO: Gene Ontology
KEGG: Kyoto Encyclopedia of Genes and Genomes
ORF: Open reading frame
PCA: Principal Components Analysis
