# Supplementary material for: Small RNA profiling for identification of microRNAs involved in regulation of seed development and lipid biosynthesis in yellowhorn
Source: BMC Plant Biol. 2021 Oct 12;21:464. doi: 10.1186/s12870-021-03239-4 (PMC8513341; doi:10.1186/s12870-021-03239-4)
Supplement: Supplementary file 1 — Additional file 1: Table S1. Distribution of small RNAs among different categories in HO yellowhorn. [file 12870_2021_3239_MOESM1_ESM.docx]

Table S1 Distribution of small RNAs among different categories in HO yellowhorn.

| Category | HO40_1 | HO40_2 | HO54_1 | HO54_2 | HO68_1 | HO68_2 | HO81_1 | HO81_2 | Average |
| --- | --- | --- | --- | --- | --- | --- | --- | --- | --- |
| Total | 15,102,556  (100%) | 14,058,247  (100%) | 13,892,050  (100%) | 16,610,901  (100%) | 11,314,611  (100%) | 8,812,929  (100%) | 11,275,850  (100%) | 8,572,830  (100%) | 12,454,997  (100%) |
| rRNA | 142,700  (0.94%) | 194,450  (1.38%) | 339,861  (2.45%) | 458,695  (2.76%) | 270,764  (2.39%) | 205,887  (2.34%) | 769,177  (6.82%) | 386,119  (4.50%) | 345,957  (2.78%) |
| tRNA | 47,450  (0.31%) | 36,431  (0.26%) | 31,141  (0.22%) | 39,088  (0.24%) | 24,539  (0.22%) | 17,451  (0.20%) | 44,915  (0.40%) | 25,979  (0.30%) | 33,374  (0.27%) |
| snoRNA | 8,868  (0.06%) | 11,366  (0.08%) | 8,403  (0.06%) | 13,980  (0.08%) | 5,739  (0.05%) | 3,447  (0.04%) | 4,551  (0.04%) | 8,736  (0.10%) | 8,136  (0.07%) |
| snRNA | 1,003  (0.01%) | 898  (0.01%) | 976  (0.01%) | 899  (0.01%) | 532  (0.00%) | 295  (0.00%) | 908  (0.01%) | 1,613  (0.02%) | 891  (0.01%) |
| Other Rfam RNA | 969  (0.01%) | 1,844  (0.01%) | 4,218  (0.03%) | 10,219  (0.06%) | 3,281  (0.03%) | 2,267  (0.03%) | 29,258  (0.26%) | 8,543  (0.10%) | 7,575  (0.06%) |
| Repeat | 1,610  (0.01%) | 2,436  (0.02%) | 2,782  (0.02%) | 8,058  (0.05%) | 1,835  (0.02%) | 2,253  (0.03%) | 5,719  (0.05%) | 2,903  (0.03%) | 3,450  (0.03%) |
| NAT | 1,238,335  (8.20%) | 1,009,924  (7.18%) | 997,023  (7.18%) | 810,490  (4.88%) | 473,177  (4.18%) | 149,976  (1.7%) | 324,831  (2.88%) | 481,353  (5.61%) | 685,639 (5.50%) |
| phasiRNA | 13,273  (0.09%) | 27,767  (0.20%) | 23,315  (0.17%) | 61,382  (0.37%) | 31,228  (0.28%) | 14,703  (0.17%) | 9,603  (0.09%) | 10,955  (0.13%) | 24,028  (0.19%) |
| miRNA | 536,363  (9.16%) | 985,956  (7.01%) | 662,046  (4.77%) | 1,439,470  (8.67%) | 593,406  (5.24%) | 280,224  (3.18%) | 276,338  (2.45%) | 259,815  (3.03%) | 629,202  (5.05%) |
| Unknown | 13,112,556  (86.82%) | 11,788,119  (83.85%) | 11,822,760  (85.10%) | 13,769,895  (82.90%) | 9,910,548  (87.59%) | 8,136,993  (92.33%) | 9,811,388  (87.01%) | 7,387,302  (86.17%) | 10,717,445  (86.05%) |
| Unique reads | 7,855,937 | 6,309,713 | 5,887,512 | 5,037,736 | 3,451,084 | 1,158,052 | 1,319,935 | 2,261,906 | 4,160,234 |
