# Supplementary material for: Small RNA profiling for identification of microRNAs involved in regulation of seed development and lipid biosynthesis in yellowhorn
Source: BMC Plant Biol. 2021 Oct 12;21:464. doi: 10.1186/s12870-021-03239-4 (PMC8513341; doi:10.1186/s12870-021-03239-4)
Supplement: Supplementary file 2 — Additional file 2: Table S2. Distribution of small RNAs among different categories in LO yellowhorn. [file 12870_2021_3239_MOESM2_ESM.docx]

Table S2 Distribution of small RNAs among different categories in LO yellowhorn.

| Category | LO40_1 | LO40_2 | LO54_1 | LO54_2 | LO68_1 | LO68_2 | LO81_1 | LO81_2 | Average |
| --- | --- | --- | --- | --- | --- | --- | --- | --- | --- |
| Total | 10,957,861  (100%) | 9,280,953  (100%) | 11,705,350  (100%) | 11,515,589  (100%) | 11,982,804  (100%) | 14,562,835  (100%) | 14,843,273  (100%) | 10,469,421  (100%) | 11,914,761  (100%) |
| rRNA | 732,062  (6.68%) | 196,101  (2.11%) | 419,531  (3.58%) | 453,234  (3.94%) | 301,916  (2.52%) | 427,752  (2.94%) | 1,865,647  (12.57%) | 1,341,042  (12.81%) | 717,161  (6.02%) |
| tRNA | 178,774  (1.63%) | 135,192  (1.46%) | 31,102  (0.27%) | 41,948  (0.36%) | 27,177  (0.23%) | 46,297  (0.32%) | 122,962  (0.83%) | 234,542  (2.24%) | 102,249  (0.86%) |
| snoRNA | 42,838  (0.39%) | 25,164  (0.27%) | 4,168  (0.04%) | 8,823  (0.08%) | 2,454  (0.02%) | 3,660  (0.03%) | 10,432  (0.07%) | 7,863  (0.08%) | 13,175  (0.11%) |
| snRNA | 1,652  (0.02%) | 742  (0.01%) | 236  (0.00%) | 525  (0.00%) | 629  (0.01%) | 858  (0.01%) | 1,431  (0.01%) | 1,667  (0.02%) | 968  (0.01%) |
| Other Rfam RNA | 5,617  (0.05%) | 3,730  (0.04%) | 2,024  (0.02%) | 2,684  (0.02%) | 2,506  (0.02%) | 4,319  (0.03%) | 60,176  (0.41%) | 33,631  (0.32%) | 14,336  (0.12%) |
| Repeat | 6,702  (0.06%) | 5,635  (0.06%) | 2,337  (0.02%) | 3,288  (0.03%) | 1,038  (0.01%) | 2,175  (0.01%) | 10,307  (0.07%) | 13,511  (0.13%) | 5,624  (0.05%) |
| NAT | 518,266  (4.73%) | 430,790  (4.64%) | 257,578  (2.20%) | 494,436  (4.29%) | 687,574  (5.74%) | 735,993  (5.05%) | 491,603  (3.31%) | 385,363  (3.68%) | 500,200  (4.20%) |
| phasiRNA | 20,293  (0.19%) | 14,592  (0.16%) | 23,171  (0.20%) | 30,839  (0.27%) | 35,750  (0.30%) | 50,178  (0.34%) | 9,471  (0.06%) | 18,105  (0.17%) | 25,300  (0.21%) |
| miRNA | 1,003,199  (9.16%) | 756,057  (8.15%) | 436,597  (3.73%) | 625,533  (5.43%) | 441,647  (3.69%) | 791,884  (5.44%) | 223,456  (1.51%) | 344,738  (3.29%) | 577,889  (4.85%) |
| Unknown | 8,449,739  (77.11%) | 7,713,913  (83.12%) | 10,529,290  (89.95%) | 9,855,039  (85.58%) | 10,482,649  (87.48%) | 12,500,558  (85.84%) | 12,050,047  (81.18%) | 8,091,227  (77.28%) | 9,959,058  (83.59%) |
| Unique reads | 3,137,598 | 2,664,102 | 1,892,087 | 3,428,275 | 3,929,011 | 4,647,773 | 1,262,957 | 1,714,044 | 2,834,481 |
