# Supplementary material for: Small RNA profiling for identification of microRNAs involved in regulation of seed development and lipid biosynthesis in yellowhorn
Source: BMC Plant Biol. 2021 Oct 12;21:464. doi: 10.1186/s12870-021-03239-4 (PMC8513341; doi:10.1186/s12870-021-03239-4)
Supplement: Supplementary file 4 — Additional file 4: Table S4. Species and abbreviations of the known miRNAs of yellowhorn. [file 12870_2021_3239_MOESM4_ESM.docx]

Table S4 Species and abbreviations of the known miRNAs of yellowhorn*.*

| Abbreviation | Species | Abbreviation | Species |
| --- | --- | --- | --- |
| aly | Arabidopsis lyrata | mes | Manihot esculenta |
| ata | Aegilops tauschii | mtr | Medicago truncatula |
| ath | Arabidopsis thaliana | nta | Nicotiana tabacum |
| bdi | Brachypodium distachyon | osa | Oryza sativa |
| bna | Brassica napus | peu | Populus euphratica |
| bol | Brassica oleracea | ppe | Prunus persica |
| bra | Brassica rapa | ptc | Populus trichocarpa |
| cme | Cucumis melo | rco | Ricinus communis |
| cpa | Carica papaya | rgl | Rehmannia glutinosa |
| csi | Citrus sinensis | sbi | Sorghum bicolor |
| ghr | Gossypium hirsutum | sly | Solanum lycopersicum |
| gma | Glycine max | ssl | Salvia sclarea |
| hbr | Hevea brasiliensis | stu | Solanum tuberosum |
| hex | Helianthus exilis | tae | Triticum aestivum |
| lja | Lotus japonicus | tcc | Theobroma cacao |
| lus | Linum usitatissimum | vvi | Vitis vinifera |
| mdm | Malus domestica | zma | Zea mays |
