# Supplementary material for: Small RNA profiling for identification of microRNAs involved in regulation of seed development and lipid biosynthesis in yellowhorn
Source: BMC Plant Biol. 2021 Oct 12;21:464. doi: 10.1186/s12870-021-03239-4 (PMC8513341; doi:10.1186/s12870-021-03239-4)
Supplement: Supplementary file 9 — Additional file 9: Table S9. Identified candidate targets for partial known and novel miRNAs. [file 12870_2021_3239_MOESM9_ESM.docx]

Table S9 Identiﬁed candidate targets for partial known and novel miRNAs.

| miRNA | Target sequence | Target gene | Target gene annotation |
| --- | --- | --- | --- |
| miR172b | TRINITY_DN25129_c2_g1 | ARF2 | Auxin response factor 2 |
| miR171i-p5_1 | TRINITY_DN29073_c1_g5 | GRF5 | Growth-regulating factor 5 |
| miR171k-5p_1 | TRINITY_DN8531_c0_g1 | ERF3 | Ethylene-responsive transcription factor 3 |
| miR7760-p3_1 | TRINITY_DN27736_c0_g1 | AGL61 | AGAMOUS-LIKE 61 |
|  | TRINITY_DN19566_c1_g1 | KAR | 3-oxoacyl-ACP reductase |
| miR319p_1 | TRINITY_DN27037_c0_g1 | FAD2-2 | Omega-6 fatty acid desaturase 2-2 |
| Xso-miRn80 | TRINITY_DN1804_c0_g1 | WRKY41 | WRKY transcription factor 41 |
| miR1536-p5_2 | TRINITY_DN25952_c0_g1 | LPAT5 | Lysophosphatidyl acyltransferase 5 |
| miR5647-p3_1 | TRINITY_DN17735_c0_g2 | DGAT1 | Diacylglycerol acyltransferase 1 |
| miR7760-p5_1 | TRINITY_DN32436_c5_g2 | MED15A | Mediator subunit 15a |
|  | TRINITY_DN15344_c0_g1 | SDR1 | Short-chain type dehydrogenase/reductase |
| miR5655-p3 | TRINITY_DN32722_c1_g1 | PCMP-H42 | Pentatricopeptide repeat-containing protein |
