# Supplementary material for: Small RNA profiling for identification of microRNAs involved in regulation of seed development and lipid biosynthesis in yellowhorn
Source: BMC Plant Biol. 2021 Oct 12;21:464. doi: 10.1186/s12870-021-03239-4 (PMC8513341; doi:10.1186/s12870-021-03239-4)
Supplement: Supplementary file 11 — Additional file 11: Figure S1. Nucleotide and deduced amino acid sequence of cDNA encoding Mediator subunit 15a (XsMED15A). The start codon is marked in red color font. An asterick represents the stop codon. [file 12870_2021_3239_MOESM11_ESM.docx]

1 ATGGATCCAGAAGAGATCTCGAGAAGGTGTGCGAAGTTGTCTCTGACAGAGGAAGACGGT

1 M D P E E I S R R C A K L S L T E E D G

61 CCTGCGGCCCAAATTGGTGCAGCATCTCTAGATTCCACAGCCCAGACAGGACATGCAAAT

21 P A A Q I G A A S L D S T A Q T G H A N

121 GGGGGTGATTGGCAAGAGGAGGCATATCAAAAGATTAAAGCCATGAAGGAGATGTATTAT

41 G G D W Q E E A Y Q K I K A M K E M Y Y

181 CCTGAATTAAATGAAATGTACCAGAAGATTACTACTAAGTTGCAGCAGCATGATTCTCTT

61 P E L N E M Y Q K I T T K L Q Q H D S L

241 CCACAACCACCAAAATCAGAGCAGCTTGAGAAGCTGAAGCTATTTAAGTCCATGTTGGAG

81 P Q P P K S E Q L E K L K L F K S M L E

301 CGTATTATAACATTCTTACAGGTTTCCAAAAATAATATCTCACCTGGTTTTAAGGACAAG

101 R I I T F L Q V S K N N I S P G F K D K

361 ATAGGTTCCTATCAGAAGCAGATAGTAAATTTCATAGCTACAAATAGGCCCAGGAAGCCT

121 I G S Y Q K Q I V N F I A T N R P R K P

421 TCTATGCAGCAAGGGCAACTTCCCCCACCTCACTTGCATCCCCTGCAGCAGCCACAATCA

141 S M Q Q G Q L P P P H L H P L Q Q P Q S

481 CAAATTTCTCAATTGCAGTCTCATGACAACCAAATGAACCCACAGATGCAATCAATGAAC

161 Q I S Q L Q S H D N Q M N P Q M Q S M N

541 TTACAAGGTTCTGTGGCAACAATGCAACAGAACAATATGACAAGTTTGCAGCACAATGTT

181 L Q G S V A T M Q Q N N M T S L Q H N V

601 ATATCTTCTATCTCAGGGGTTTCAACAGCACAGCAGAACATGTTAAATTCATTGCAGCCT

201 I S S I S G V S T A Q Q N M L N S L Q P

661 GGTTCCAATTTGGATTCAGGACAAGGAAACACAATGAACTCCTTGCAGCAGGTTGCTGTT

221 G S N L D S G Q G N T M N S L Q Q V A V

721 GGATCACTGCCACAAAATTCTGTCAGTGCTTCCCAGCAAGCAAACATTAATACTTTGTCT

241 G S L P Q N S V S A S Q Q A N I N T L S

781 TCACAAAGTGGAGTTAATATGCTCCAGTCAAATATTAATCCCCTCCAGTCAAACTCCAAT

261 S Q S G V N M L Q S N I N P L Q S N S N

841 ATGCTGCAACACCAGCATTTGAAACATCAGGAGCAGCAAATGTTGCAATCACAACAGCTC

281 M L Q H Q H L K H Q E Q Q M L Q S Q Q L

901 AAACAACAAATTCAGCAGCGCCAGATACAGCAGCAATTAATGCAGAAGCAGCAGCAGCAG

301 K Q Q I Q Q R Q I Q Q Q L M Q K Q Q Q Q

961 CAATTGCATCAGCAACAACATCAGCAATTGCACCAGCAGGCCAAGCAACAGCTTCCTGCA

321 Q L H Q Q Q H Q Q L H Q Q A K Q Q L P A

1021 CAGTTGCAGACACACCAAATGCAGCAGCTGCATCAGATGAATGACGTAGGTGACTTGAAG

341 Q L Q T H Q M Q Q L H Q M N D V G D L K

1081 ATGAGACAGAGTATGGGTGTTAAGCCAGGGGTCTTTCAACAACATCTGTCTTCAAGCCAG

361 M R Q S M G V K P G V F Q Q H L S S S Q

1141 CGCCCAGCTTATCCCCATCAACAGTTGAAACCTGGAGCTACATTTCCTATTTCCTCACCT

381 R P A Y P H Q Q L K P G A T F P I S S P

1201 CAACTCCTTCAGGCTGCTTCCCCTCAAATGCCACAACATTCTTCTCCACAGATCGACCAA

401 Q L L Q A A S P Q M P Q H S S P Q I D Q

1261 CAAAATTTGCTCTCATCACTCCCCAAAACTGGAACCCCATTGCAATCTGCTAACTCTCCA

421 Q N L L S S L P K T G T P L Q S A N S P

1321 TTTGTTGTTCCATCTCCTTCAACACCCTTGGCTCCGTCCCCTATGCCAGGAGATTCTGAA

441 F V V P S P S T P L A P S P M P G D S E

1381 AAACCCATTTCCGGTATGTCCTCACTCTCAAATGCTGGAAACATTGGACATCAGCAAACC

461 K P I S G M S S L S N A G N I G H Q Q T

1441 ACTAGTGCACAAGCAGCAGCTCCATCCCTTGCAATCGGTACTCCTGGGATATCAGCCTCA

481 T S A Q A A A P S L A I G T P G I S A S

1501 CCTTTGCTTGCAGAGTTTACTGGTCCAGATGGTACTCATGCTACTGCCTTGGCAGCTGTT

501 P L L A E F T G P D G T H A T A L A A V

1561 CCCGGCAAGTCGAGTGTTACAGAACAGCCCCTTGAGCGCTTAATTAAAGCAGTGAAATCA

521 P G K S S V T E Q P L E R L I K A V K S

1621 ATGTCTTCGAATGCACTGAGTGCATCTGTCAGTGACATTGGTTCAGTTGTGAGTATGATT

541 M S S N A L S A S V S D I G S V V S M I

1681 GATAGGATTGCTGGATCAGCACCAGGTAATGGGTCTAGAGCTGCAGTTGGTGAAGATTTG

561 D R I A G S A P G N G S R A A V G E D L

1741 GTTGCCATGACCAAGTGTCGTCTGCAAGCTAGAAATTTTATCATACAAGATGGAATGACT

581 V A M T K C R L Q A R N F I I Q D G M T

1801 GGACCTCGGAAAATTAGGCGCTACACAAGTGCCATGCCCTTAAATGTAGTATCATCAGCT

601 G P R K I R R Y T S A M P L N V V S S A

1861 GGCAGCATGAGTGACAGTAGTTTCAAGCAGTTGATTGGTGTGGAGACATCTGATCTAGAG

621 G S M S D S S F K Q L I G V E T S D L E

1921 TCAACTGCAACATCTAGTATCAAGAGGCCAAGAGTAGAGGCTAATCATGTCCTTTCGGAA

641 S T A T S S I K R P R V E A N H V L S E

1981 GAAATAAGGGAAATAAATCAACAACTTATAGACACGGTGGTAGATATTAGTGACGAAGAC

661 E I R E I N Q Q L I D T V V D I S D E D

2041 GTTGATCCGACTGCGGCTGCTGCTGCTGCTGCTGAAGGGGGTGAAGGAACCATTGTTAAG

681 V D P T A A A A A A A E G G E G T I V K

2101 TGCTCTTTCAATGCTGTGGCTCTCAGTCGGCACTTAAAATCACAGTATGATTCAGCACAA

701 C S F N A V A L S R H L K S Q Y D S A Q

2161 ATGTCACCTATTCAGCCCTTGCGGTTGCTTGTTCCAACAAATTACCCAAATTGCTCTCCA

721 M S P I Q P L R L L V P T N Y P N C S P

2221 ATGCTATTAGACAAATTTCCAGTTGAAGTCAGTAAGGAATATGAAGATGTTTCTGTGAAA

741 M L L D K F P V E V S K E Y E D V S V K

2281 GCAAAGTCAAGGTTTAGTATATCCCTGCGGAGCCTTTCGCAACCTATGTCACTTGGGGAT

761 A K S R F S I S L R S L S Q P M S L G D

2341 ATAGCAAGGACCTGGGATGTTTGCGCACGAGCAGTTATTTCCGAGTATGCACAGCAGAGC

781 I A R T W D V C A R A V I S E Y A Q Q S

2401 GGTGGAGGCAGTTTCAGCTCTAAATATGGGACTTGGGAAAACTGCTTGAGTGCTGCAATC

801 G G G S F S S K Y G T W E N C L S A A I

2461 TGA

821 *
