# Supplementary figures and images for: Small RNA profiling for identification of microRNAs involved in regulation of seed development and lipid biosynthesis in yellowhorn
Source: BMC Plant Biol. 2021 Oct 12;21:464. doi: 10.1186/s12870-021-03239-4 (PMC8513341; doi:10.1186/s12870-021-03239-4)

## Slide 1
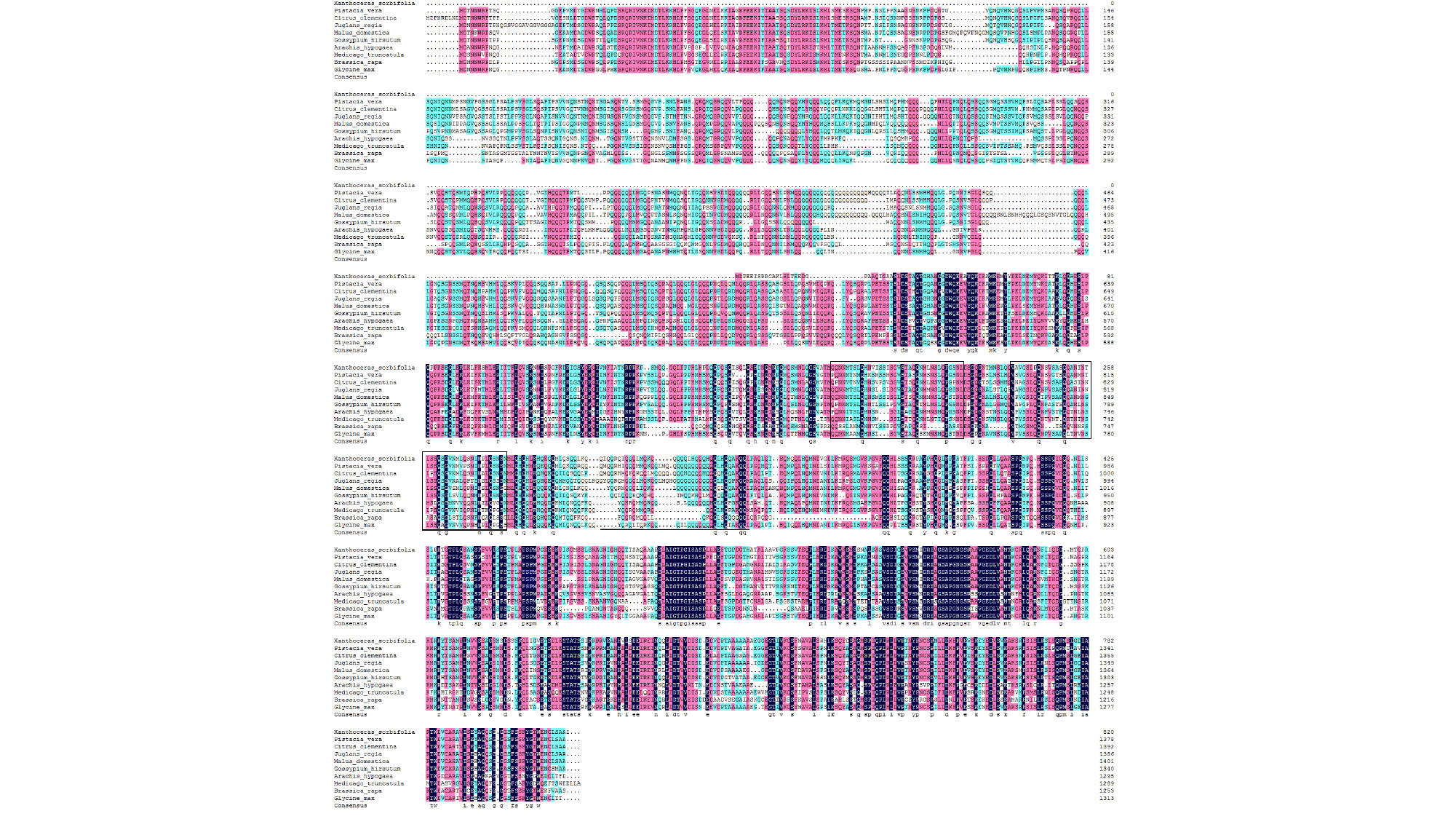

Supplement: Supplementary file 12 — Additional file 12: Figure S2. Multiple sequence alignment of Mediator subunit 15a from different species. The MED15A protein sequences were chosen from different species and aligned by DNAMAN. The two internal repeats within XsMED15A are shown in black boxes. The predicted coiled coil region is underlined. GenBank accession numbers of MED15A protein sequences used for the comparison are showed as follows: Xanthoceras sorbifolium (BankIt2507240 XsMED15A OK432284), Pistacia vera (XP_031251638.1), Citrus clementina (XP_024036133.1), Juglans regia (XP_035548095.1), Malus domestica (XP_028955279.1), Gossypium hirsutum (XP_040969330.1), Arachis hypogaea (XP_025617476.1), Medicago truncatula (XP_024636817.1), Brassica rapa (XP_033134666.1), and Glycine max (XP_003547623.1). [file 12870_2021_3239_MOESM12_ESM.pptx]
