# Supplementary material for: Small RNA profiling for identification of microRNAs involved in regulation of seed development and lipid biosynthesis in yellowhorn
Source: BMC Plant Biol. 2021 Oct 12;21:464. doi: 10.1186/s12870-021-03239-4 (PMC8513341; doi:10.1186/s12870-021-03239-4)
Supplement: Supplementary file 13 — Additional file 13: Table S11. Primers for qRT-PCR analysis of differentially expressed miRNAs. [file 12870_2021_3239_MOESM13_ESM.docx]

Table S11 Primers for qRT-PCR analysis of differentially expressed miRNAs.

| miRNA | Primer sequences (5'-3') |
| --- | --- |
| miR172b | AGAATCTTGATGATGCTGCAT |
| miR171i-p5_1 | CTTTTCCTTCTTCTTCTTGC |
| miR7760-p3_1 | GGAGGCGGTGGAGGTGGA |
| miR319p_1 | TTGGATTGAAGGGAGCTCC |
| Xso-miRn80 | TTGGTGTTCTTGGTGGAGATC |
| 5.8S rRNA | GTCTGCCTGGGTGTCACGCAA |
