# Supplementary material for: Small RNA profiling for identification of microRNAs involved in regulation of seed development and lipid biosynthesis in yellowhorn
Source: BMC Plant Biol. 2021 Oct 12;21:464. doi: 10.1186/s12870-021-03239-4 (PMC8513341; doi:10.1186/s12870-021-03239-4)
Supplement: Supplementary file 14 — Additional file 14: Table S12. Primers for qRT-PCR analysis of target genes of differentially expressed miRNAs. [file 12870_2021_3239_MOESM14_ESM.doc]

Table S12 Primers for qRT-PCR analysis of target genes of differentially expressed miRNAs.

| Targets | Primer sequences (5'-3') | |
| --- | --- | --- |
|  | primer F | primer R |
| ARF2 | CGGAGCAGGAGACACAGAAA | CAAGGTGAGGTGGGATGAAAC |
| GRF5 | AGAAGAGGAGGAGGAAGAAGAG | AGGTGCAGAAGAACAGATGG |
| AGL61 | AGAAAGGTTGATCAGGCGATG | GAGGTGGAGAGGGTGTTGGA |
| KAR | CGCTGCTGACGAATTGATG | ACGGCAAAGTGGTGATGGT |
| FAD2 | GGCAGCGATGGATGAGTGT | GGCTGGCTTGGCTTGTATG |
| WRKY41 | CCTCCCTATTGTCCAAATCCTCT | GCATCACCATCAGTGTCCAAA |
| LPAT5 | TGTGGAGTACCACCATCATCAAC | AGGTGAAGGAAAAGGAGAAGACAA |
| DGAT1 | GCCATGTAACTCCCAGAGTCATAA | GCCTGTTCATAAATGGATGGTTC |
| MED15A | GCAGGACCGTCTTCCTCTGT | GGTTGCGATTGGGGATTTT |
| β-actin | TACCGAGGCACCATTAAATCCC | AAGGTCCAAACGAAGAATAGCA |
